# Phylogenetic diversity in freshwater‐dwelling Isochrysidales haptophytes with implications for alkenone production

**DOI:** 10.1111/gbi.12330

**Published:** 2019-02-05

**Authors:** Nora Richter, William M. Longo, Sarabeth George, Anna Shipunova, Yongsong Huang, Linda Amaral‐Zettler

**Affiliations:** ^1^ Department of Earth, Environmental and Planetary Sciences Brown University Providence Rhode Island; ^2^ The Josephine Bay Paul Center for Comparative Molecular Biology and Evolution Marine Biological Laboratory Woods Hole Massachusetts; ^3^ Department of Marine Chemistry and Geochemistry Woods Hole Oceanographic Institution Woods Hole Massachusetts; ^4^ Department of Marine Microbiology and Biogeochemistry NIOZ Royal Netherlands Institute for Sea Research and Utrecht University Texel The Netherlands; ^5^ Department of Freshwater and Marine Ecology Institute for Biodiversity and Ecosystem Dynamics University of Amsterdam Amsterdam Netherlands

**Keywords:** alkenones, freshwater lakes, Group I, haptophyte, Isochrysidales, phylogenetics

## Abstract

Members of the order Isochrysidales are unique among haptophyte lineages in being the exclusive producers of alkenones, long‐chain ketones that are commonly used for paleotemperature reconstructions. Alkenone‐producing haptophytes are divided into three major groups based largely on molecular ecological data: Group I is found in freshwater lakes, Group II commonly occurs in brackish and coastal marine environments, and Group III consists of open ocean species. Each group has distinct alkenone distributions; however, only Groups II and III Isochrysidales currently have cultured representatives. The uncultured Group I Isochrysidales are distinguished geochemically by the presence of tri‐unsaturated alkenone isomers (C_37:3b_ Me, C_38:3b_ Et, C_38:3b_ Me, C_39:3b_ Et) present in water column and sediment samples, yet their genetic diversity, morphology, and environmental controls are largely unknown. Using small‐subunit (SSU) ribosomal RNA (rRNA) marker gene amplicon high‐throughput sequencing of environmental water column and sediment samples, we show that Group I is monophyletic with high phylogenetic diversity and contains a well‐supported clade separating the previously described “EV” clade from the “Greenland” clade. We infer the first partial large‐subunit (LSU) rRNA gene Group I sequence phylogeny, which uncovered additional well‐supported clades embedded within Group I. Relative to Group II, Group I revealed higher levels of genetic diversity despite conservation of alkenone signatures and a closer evolutionary relationship with Group III. In Group I, the presence of the tri‐unsaturated alkenone isomers appears to be conserved, which is not the case for Group II. This suggests differing environmental influences on Group I and II and perhaps uncovers evolutionary constraints on alkenone biosynthesis.

## 1 INTRODUCTION

Quantitative estimates of past terrestrial temperatures are essential for testing and developing climate models that extend past the historical record to assess regional temperature variations (Otto‐Bliesner et al., 2016). Lake sediment archives are sensitive to continental‐scale, local, and regional climate variations and are often ideal for the preservation of organic proxies as recorders of terrestrial temperature changes (Castañeda & Schouten, 2011). Alkenones, for instance, are long‐chain methyl and ethyl ketones (C_35_–C_42_) that show potential as lake surface temperature proxies (Castañeda & Schouten, 2011; Chu et al., 2005; Longo et al., 2016; Sun, Chu, Liu, Li, & Wang, 2007; Theroux, Toney, Amaral‐Zettler, & Huang, 2013; Toney et al., 2012; Zink, Leythaeuser, Melkonian, & Schwark, 2001). These compounds were first identified in ocean sediments (de Leeuw, van der Meer, Rijpstra, & Schenck, 1980), and it was later noted that varying degrees of alkenone unsaturation were correlated with fluctuations in sea surface temperatures (Brassell, Eglinton, Marlowe, Pflaumann, & Sarnthein, 1986; Prahl & Wakeham, 1987). Haptophyte alkenone producers belong to the order Isochrysidales and are divided into three major groups: Group I freshwater species, Group II brackish/estuarine species, and Group III open ocean species (D'Andrea, Theroux, Bradley, & Huang, 2016; Theroux, D'Andrea, Toney, Amaral‐Zettler, & Huang, 2010).

Previous studies identified two main alkenone producers in the open ocean: *Gephyrocapsa oceanica* (Conte, Thompson, Eglinton, & Green, 1995; Volkman, Barrett, Blackburn, & Sikes, 1995) and *Emiliania huxleyi* (Conte et al., 1995; Volkman, Eglinton, Corner, & Sargent, 1980). A combination of ocean surface sediment and sediment trap calibration studies (Brassell et al., 1986; Prahl, Muehlhausen, & Zahnle, 1988; Prahl & Wakeham, 1987) and culture studies (Conte, Thompson, Lesley, & Harris, 1998; Conte et al., 1995) were used to demonstrate that alkenone production by Group III Isochrysidales corresponds to sea surface temperature changes. Understanding how alkenone production in lakes relates to temperature changes, however, is more complex for three main reasons: (a) lake environments tend to be more chemically diverse and are more susceptible to varying regional environmental and climatic factors (Castañeda & Schouten, 2011), this can drive differences in haptophyte productivity, alkenone production, and potentially, species variability; (b) species mixing between Group I and II phylotypes (Theroux et al., 2010) and even within the Group II phylotypes, is a known problem in many lakes and can influence temperature reconstructions (Randlett et al., 2014; Toney et al., 2010, 2012); and lastly, (c) while there are cultures available for Group II Isochrysidales (Sun et al., 2007; Theroux et al., 2013; Toney et al., 2012; Zheng, Huang, Andersen, & Amaral‐Zettler, 2016), there are no available cultures for Group I haptophytes, complicating our ability to isolate and determine environmental controls on Group I alkenone temperature responses.

Recent environmental studies show that we can distinguish Group I alkenone profiles from Group II and III by the identification of tri‐unsaturated isomeric alkenones (Longo, Dillon, Tarozo, Salacup, & Huang, 2013; Longo et al., 2016). Group I alkenones are present in numerous freshwater, alkaline lakes across the Northern Hemisphere (Longo et al., 2018); however, little is known about the haptophyte producers since they have never been physically described or isolated in pure culture.

The phylum Haptophyta and the order Isochrysidales are very diverse and occur in a range of environments (Bendif, Probert, Schroeder, & de Vargas, 2013; Edvardsen, Egge, & Vaulot, 2016; Egge et al., 2013; Gran‐Stadniczeñko, Šupraha, Egge, & Edvardsen, 2017; Liu et al., 2009); however, few studies have focused on the diversity of Group I Isochrysidales. Previous studies confirmed that multiple Group I operational taxonomic units (OTUs) occur in the same lake (D'Andrea et al., 2006, 2016; Theroux et al., 2010), and a later study further recommended the establishment of a new clade within Isochrysidales called Group “EV” (Simon, López‐García, Moreira, & Jardillier, 2013). Here, we show that the shared recent common ancestry of the “Greenland phylotype” and “EV” is strongly supported, and we also provide evidence for additional well‐supported branching patterns within the Group I. We focus on this diversity in a suite of Alaskan lakes, but then extend this comparison to lakes in Germany and Iceland to demonstrate the broader applications of our findings (Supporting Information, Figure S1). These findings describe the genetic diversity of the monophyletic Group I haptophytes, thereby guiding future interpretations of their distinct biomarker distributions in lacustrine sedimentary records.

## 2 RESULTS

### 2.1 Haptophyte small‐subunit (HSSU) rRNA gene oligotypes

**Figure 1:**
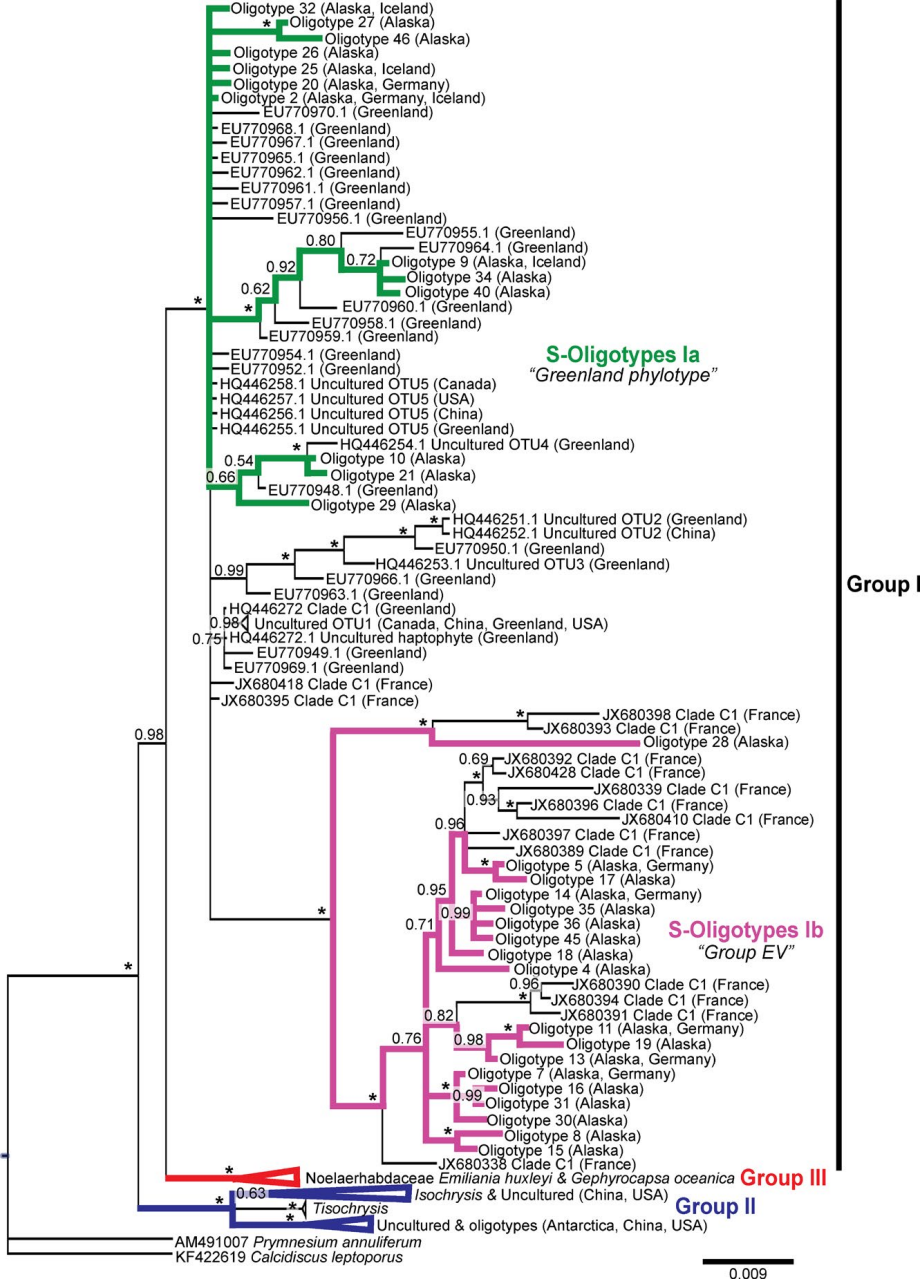
Haptophyte small‐subunit phylogenetic tree with oligotypes analyzed in this study as indicated by the green and pink colored branches, corresponding to S‐Oligotypes Ia (“Greenland phylotype”) and S‐Oligotypes Ib (“Group EV”), respectively. The posterior probability for each node is indicated, where “*” corresponds to a probability of 1.0. Note Groups I and III are closely related with 0.98 branch support, relative to Group II. An expanded version of the tree can be found in the Supporting Information Figure S6 [Colour figure can be viewed at wileyonlinelibrary.com]

**Figure 2:**
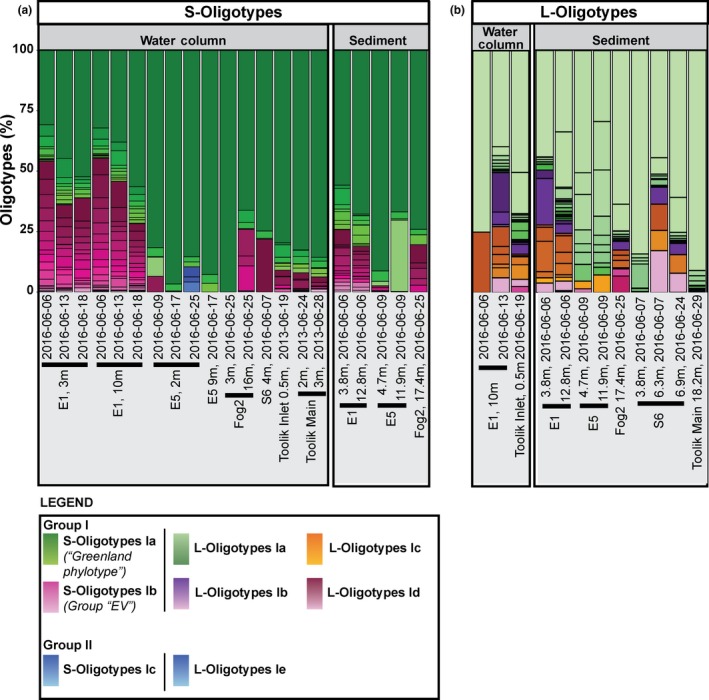
This figure shows the relative read abundance of (a) haptophyte small‐subunit and (b) haptophyte large‐subunit (HLSU) oligotypes for samples from Alaska analyzed in this study. (a) The green shades correspond to S‐Oligotypes Ia, whereas the maroon shades correspond to S‐Oligotypes Ib. (b) This figure reflects the relative read abundance of HLSU oligotypes for different samples analyzed in this study, where light green, purple, orange, and pink correspond to L‐Oligotypes Ia, Ib, Ic, and Id, respectively. Group II oligotypes are shown in blue for both S‐Oligotypes and L‐Oligotypes. Group II L‐Oligotypes were found in Toolik Lake; however, only two sequences were recovered, one for L‐Oligotype 2 and another for L‐Oligotype 3 [Colour figure can be viewed at wileyonlinelibrary.com]

**Figure 3:**
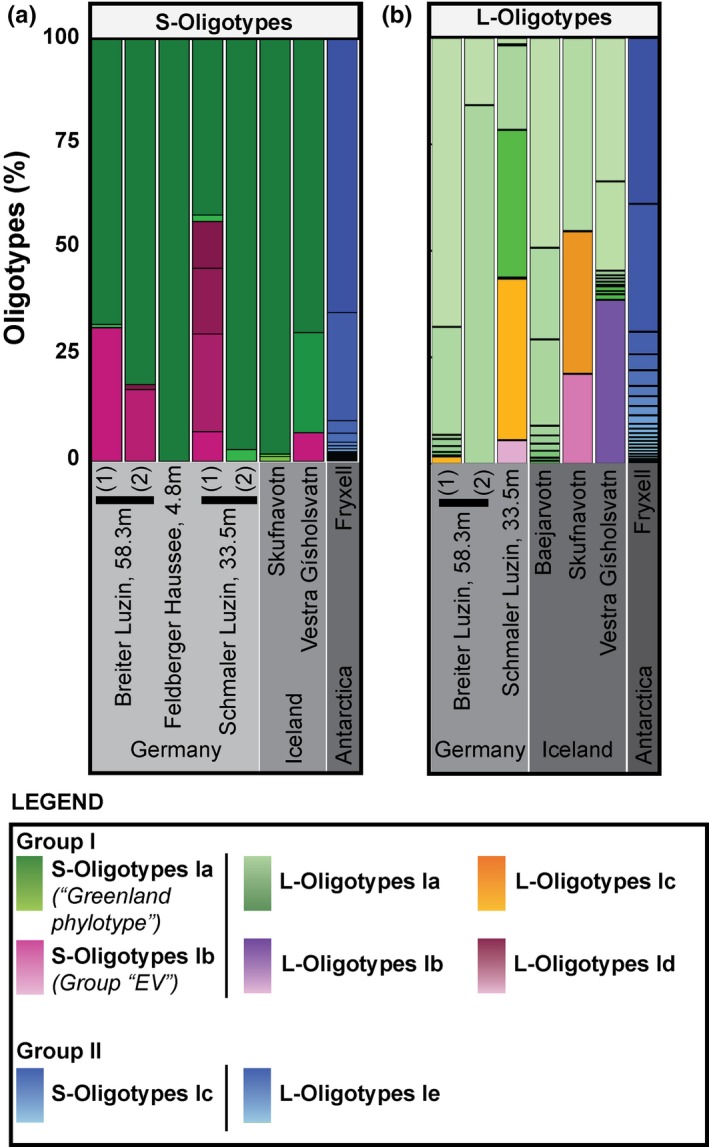
This figure reflects the relative read abundance of (a) haptophyte small‐subunit and (b) haptophyte large‐subunit (HLSU) oligotypes analyzed in this study from lakes in Germany, Iceland, and Antarctica. (a) The green shades correspond to S‐Oligotypes Ia, whereas the maroon shades correspond to S‐Oligotypes Ib. (b) This figure reflects the relative read abundance of HLSU oligotypes for different samples analyzed in this study, where light green, purple, orange, and pink correspond to L‐Oligotypes Ia, Ib, Ic, and Id, respectively. Group II oligotypes are shown in blue for both S‐Oligotypes and L‐Oligotypes [Colour figure can be viewed at wileyonlinelibrary.com]

Haptophyte‐specific primers targeting the small‐subunit rRNA gene (hereon designated haptophyte small‐subunit [HSSU]; Egge et al., 2013) followed by oligotyping revealed 31 out of 50 distinct oligotypes for Group I Isochrysidales (designated as S‐Oligotypes from hereon; Figure 1) that we further divided into S‐Oligotypes Ia and Ib. We recovered relatively high read counts for Group I even though the HSSU primers we employed also amplified non‐haptophyte species including a large number of fungi in each of our samples. The number of non‐haptophyte sequences recovered was higher in the sediment samples (91.0 ± 25.5%) than the water column samples (77.7 ± 11.8%). The greatest number of Isochrysidales identified oligotypes was present in the water column (0.1%‐27.8% Isochrysidales sequences) and sediment samples (0.5%‐1.5% Isochrysidales sequences) from Alaska (mainly Lakes E1 and E5; Figure 2a). We also recovered Group I oligotypes from surface sediment samples from German and Icelandic lakes (0.1%‐1.0% Isochrysidales sequences; Figure 3a), despite these samples being collected for alkenone characterization and not molecular ecology work. In almost all of our samples, we note a predominance of S‐Oligotypes Ia (making up 43.2%‐100.0% of the Isochrysidales sequences recovered) over S‐Oligotypes Ib (ranging from 0.0%‐56.8% for all the Isochrysidales sequences recovered). In the Alaskan water column samples (i.e., E1, E5, Fog2, and Toolik; Figure 2), we observed a seasonal succession of different oligotypes during the month of June as lake ice‐out, isothermal mixing, and incipient stratification occurred. We also observed a change in Isochrysidales community composition with water column depth (i.e., E1, E5, and Fog2).

Phylogenetic inference revealed significant branch support (posterior probability, PP = 1.0) for the monophyly of Group I, Group II (PP = 1.0), and Group III (PP = 1.0) Isochrysidales clades (Figure 1), independently. We noted that S‐Oligotypes Ia were closely related to previously defined OTUs in freshwater and brackish lakes from Canada, China, France, Greenland, and the United States (D'Andrea et al., 2006; Simon et al., 2013; Theroux et al., 2010). In particular, S‐Oligotype 2 in the S‐Oligotype Ia group was closely related to previously defined OTU 1 (Theroux et al., 2010; Figure 1) and was present in all of the HSSU‐amplified samples (except the Lake Fryxell sample, which contained only Group II sequences; Figures 1 & 3a). In addition within Group I, we noted the presence of a distinct clade (PP = 1.0) that consisted of oligotypes from this study and previous sequences from Lakes Etang des Valles (“EV”) and Annecy in France (Simon et al., 2013). Our HSSU tree also showed robust support for recent shared ancestry of Groups I and III, which differs from previous studies that suggest more recent common ancestry between Groups I and II (Gran‐Stadniczeñko et al., 2017; Simon et al., 2013; Theroux et al., 2010).

### 2.2 Haptophyte large‐subunit rRNA gene oligotypes

**Figure 4:**
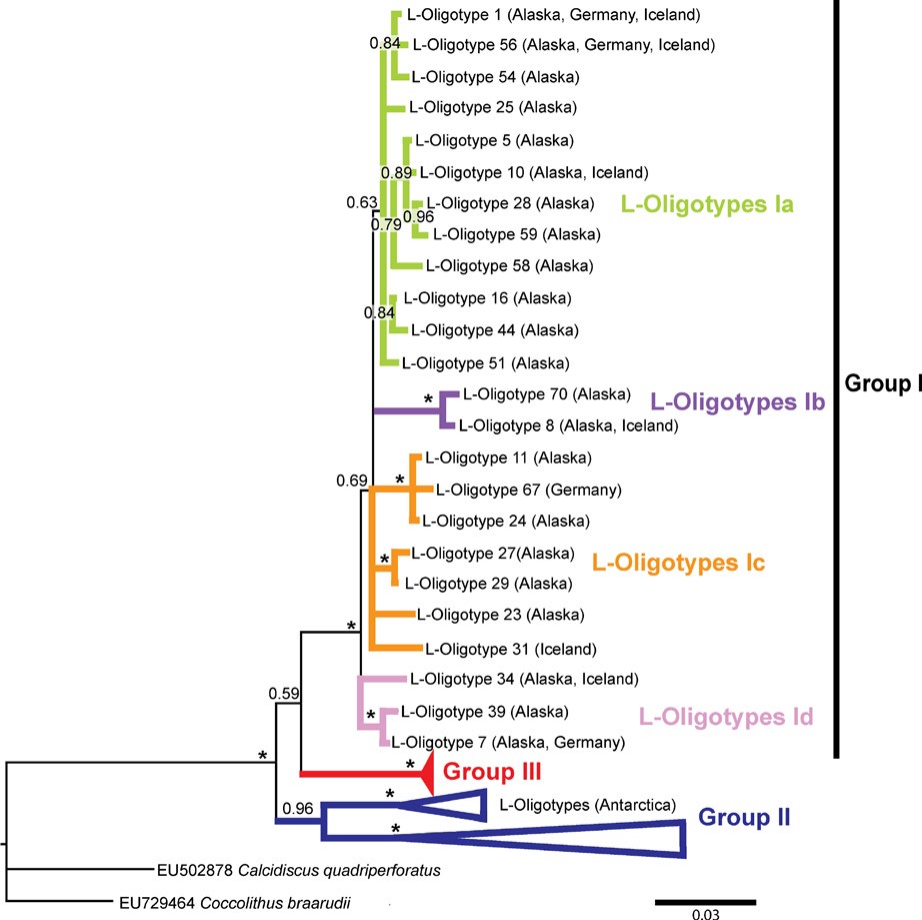
Haptophyte large‐subunit (HLSU) phylogenetic tree with oligotypes analyzed in this study indicated by the light green, purple, orange, and pink branches (note that the different colors do not correspond to the haptophyte small‐subunit oligotypes). The posterior probability for each node is indicated, where “*” corresponds to 1.0. An expanded version of the tree can be found in the supporting information, Figure S7 [Colour figure can be viewed at wileyonlinelibrary.com]

We report the first haptophyte‐specific large‐subunit rRNA gene (hereon designated haptophyte large‐subunit [HLSU]; Egge et al., 2013) sequences for the Group I clade (Figure 4). We detected 50 out of 72 oligotypes for the LSU rRNA Group I clade (referred to as L‐Oligotypes from hereon) with several subgroupings, which we define here as L‐Oligotypes Ia, Ib, Ic, and Id. Despite low total sequence counts, our trees demonstrated significant support for the monophyly of the Group I clade (PP = 1.0). We noted a general predominance of L‐Oligotypes Ia (relative recovery of 43.4%–100% of the total Isochrysidales sequences) over L‐Oligotypes Ib, Ic, and Id (0%–54.6% out of the total Isochrysidales sequences; Figures 2b & 3b).

### 2.3 Group I alkenones

We selected sampling locations based on the previous detection of Group I alkenone signatures. All alkenone profiles analyzed for this study contained the distinct C_37:3b_Me alkenone that is the hallmark of Group I Isochrysidales (Longo et al., 2016, 2018). The ratio of isomeric ketones, RIK_37_ index, which is based on changes in the fractional abundance of the C_37:3a_Me compared to the C_37:3b_Me alkenones was used to test for Group I and II mixing (Longo et al., 2016). In our study, RIK_37_ values ranged from 0.48 to 0.64, corroborating the HSSU and HLSU datasets that identified Group I as the dominant Isochrysidales haptophyte in all of these systems. The $U_{37}^{K}$ index for the Lake E1 water column samples showed a linear correlation with temperature ($U_{37}^{K}$ = 0.015T–0.64, *r* ^2 ^= 0.84, *p* < 0.05; Supporting Information, Figure S2). Alkenone fractional abundances were visualized using a heatmap (Supporting Information, Figure S3). Overall, alkenone distributions were consistent among samples. C_37_Me alkenone distributions were highly conserved with slightly greater variation among the C_38_Et, C_38_Me, and C_39_Et alkenones.

## 3 DISCUSSION

### 3.1 Group I diversity inferred from SSU and LSU rRNA gene oligotyping and phylogenetic tree reconstructions

We found Group I oligotypes (HSSU and HLSU) in samples from freshwater lakes with Group I alkenone signatures in Alaska, Germany, and Iceland (Figure 1). We demonstrated robust support for the monophyly of the Group I assemblage that includes members of the “Greenland phylotypes” and previously described Group “EV” by Simon et al. (2013). Because no LSU data existed for this clade, we were unable to determine its phylogenetic position in our HLSU phylogenetic trees (Edvardsen et al., 2016). During our alignments of all existing Group I SSU rRNA gene sequences, however, we determined that Group “EV” sequences were likely not identified in previous haptophyte molecular diversity studies that targeted the region between 427–889 bp (D'Andrea et al., 2006, 2016; Theroux et al., 2010; Toney et al., 2010) because the primers used in those studies (Coolen et al., 2009) are not compatible with Group “EV” amplification due to primer mismatch. In addition, our high‐throughput amplicon sequencing in combination with oligotyping allowed us to amplify sequences present in relatively low abundance and identify exact nucleotide variants in our sequences (Callahan, McMurdie, & Holmes, 2017; Eren, Borisy, Huse, & Mark Welch, 2014; Eren et al., 2013). This was not possible using earlier techniques that were based on clustering approaches in combination with clone library and second‐generation (e.g., capillary) sequencing technology (Callahan et al., 2017; Eren et al., 2013, 2014). Therefore, the Group “EV” clade is likely present in other lakes that were previously analyzed (e.g., lakes reported by Theroux et al., 2010). Future analyses of fresh and appropriately preserved samples from these lakes for molecular ecology work might reveal an even higher diversity of oligotypes (Willerslev & Cooper, 2005) than we present in this study.

Within the HLSU data, we found several dominant oligotypes, whereas there was only one dominant oligotype in the HSSU data: S‐Oligotype 2 in the S‐Oligotype Ia group. This is likely the result of inherently higher genetic variability in the LSU rRNA gene relative to the SSU rRNA gene due to higher mutation rates in LSU rRNA genes (Bittner et al., 2013) and therefore might provide additional insights into Group I diversity. We did observe a higher number of oligotypes within the HLSU dataset, despite the small number of samples. However, low branch support within the HLSU Group I suggested that better taxon sampling is needed before we are able to identify L‐Oligotypes that correspond to distinct S‐Oligotype clades within Group I. To test for congruence between the HSSU and HLSU oligotypes, we would need to sequence the region that spans both subunits, which was beyond the scope of this study but the subject of future efforts.

Our HSSU and HLSU results revealed another significant finding: Group I and III are more closely related than previous studies have demonstrated. Previous work reported that Groups I and II were more closely related (Gran‐Stadniczeñko et al., 2017; Simon et al., 2013; Theroux et al., 2010). This provides insight into why there is such conservation in the alkenones of Groups I and III in contrast to Group II. Group I Isochrysidales are found in a range of environmental conditions: pH levels 5.9–9.4, salinities ranging from 0 to 4.43 g/L, alkalinity from 104 to 1,976 mEq/L, and water temperatures from 0–4°C to 12–16°C (D'Andrea & Huang, 2005; D'Andrea et al., 2016; Longo et al., 2016, 2018; Plancq et al., 2018; Theroux et al., 2010; Toney et al., 2010). Group II is found in a similarly broad range of environmental conditions: pH levels 7.3 to 10.5, salinities ranging from 0.05 to 270 g/L, and water temperatures from 8°C to 28°C (Chu et al., 2005; Liu et al., 2011; Longo et al., 2016, 2018; Plancq et al., 2018; Randlett et al., 2014; Sun et al., 2007; Theroux et al., 2010; Toney et al., 2010). We observed high genetic diversity in both Groups I and II Isochyrsidales (Bendif et al., 2013; Edvardsen et al., 2016; Egge et al., 2013; Gran‐Stadniczeñko et al., 2017), but we see differing alkenone conservation between Group I and Group II (D'Andrea et al., 2016; Longo et al., 2016, 2018; Randlett et al., 2014; Theroux et al., 2010; Toney et al., 2010; Zheng et al., 2016). In Group I, we see a conservation of tri‐unsaturated alkenone isomers (C_37:3b_ Me, C_38:3b_ Et, C_38:3b_ Me, C_39:3b_ Et; Longo et al., 2016, 2018), whereas Group II culture studies point to the consistent absence of C_38_ Me alkenones (Nakamura, Sawada, Araie, Suzuki, & Shiraiwa, 2014; Ono, Sawada, Shiraiwa, & Kubota, 2012; Rontani, Beker, & Volkman, 2004; Sun et al., 2007; Theroux et al., 2013; Zheng et al., 2016). In contrast, we observe low genetic and alkenone diversity in Group III Isochyrsidales (Bendif et al., 2014; Conte et al., 1995, 1998, 2006). The low alkenone diversity in Groups I and III might point to similar mechanisms in streamlining their alkenone biosynthetic pathways, as they are more closely related to each other than to Group II Isochyrsidales (Figures 1 and 4).

### 3.2 Alaska case study: Group I oligotype succession in June

During the month of June, Lake E1 water column samples were collected during partial ice‐cover, isothermal mixing, and summer stratification. Our findings demonstrate that Group I species composition varies throughout the season (Figure 2a). In Lake E1, at both 3‐ and 10‐m depths we observed a higher number of oligotypes and relative read abundance for S‐Oligotypes Ib in the early part of the season. We recovered the largest number of reads in the early part of the season, and we recovered fewer reads and observed an increase in S‐Oligotype Ia and a decrease in S‐Oligotype Ib relative read abundance as Lake E1 underwent isothermal mixing (June 6) and summer stratification (starting June 13 and completing stratification June 18). The temporal decreases in alkenone unsaturation in our dataset corresponded to changing lake water temperatures at 3‐ and 10‐m depths, which were both in the euphotic zone (Supporting Information, Figure S4).

In Lake E5, however, we did not see a similar change in HSSU oligotypes (Figure 2a), but we did see a decrease in the number of Group I oligotypes present in the lake, and the limited appearance of Group II oligotypes on June 25 at 2‐m depth. In comparison with Lake E1, Lake E5 appears to have a longer isothermal mixing period and remained partially ice‐covered until June 21 (Supporting Information, Figure S5).

We observed a comparatively high number of oligotypes present in the E1 datasets for HSSU and HLSU (Figure 2a,b). This was not apparent in the other Alaskan lakes, which had a lower number of oligotypes present in both the water column and surface sediment samples. In addition, we did not observe a major difference in the oligotypes found in the water column and surface sediment samples (Figure 2a). This suggests that the oligotypes we observed in the water column were getting preserved in the sediment. This also suggests that we were sampling the water column during the peak of the Group I Isochrysidales bloom. It should be noted that we observed a predominance of S‐Oligotypes Ia in all of the sediment samples from Alaska (i.e., E1, E5, and Fog2; Figure 2a). Similarly, we saw a predominance of L‐Oligotypes Ia in all of the surface sediment samples from Alaska (i.e., E1, E5, Fog2, S6, and Toolik; Figure 2b).

### 3.3 Conservation of alkenone signatures

Despite the large genetic diversity in Group I Isochrysidales, previous work suggested that variations in alkenone composition were due to temperature and that the high number of oligotypes did not play a major role in alkenone biomarker applications in these lakes (Longo et al., 2016, 2018). In Lake E1 from Alaska, for instance, we quantified alkenone concentrations and sequenced DNA from the same water column samples. In our sequence data for HSSU, we observed extensive diversity in Group I throughout the month of June (Figure 2a), but we still observed a correlation between alkenones and temperature (Longo et al., 2016; Supporting Information, Figure S2). The largest variation that we observed in the alkenone profiles was in C_37:4_Me, which corresponded to changes in lake water temperature.

Considering alkenone profiles from sediments for all of the sites analyzed in this study (Supporting Information, Figure S3), we saw that variability in the C_37_Me group was similar for sediment samples from the same temperature regime (note that Iceland samples from Baejarvotn and Skufnavotn were from a similar temperature regime, while samples from Vestra Gíslholtsvatn were from a warmer region). This corroborates a recent finding that C_37_Me alkenone production in response to temperature may be consistent among Group I Isochrysidales (Longo et al., 2018). In our study, we only present a small range of lakes that are typically cold biased. However, as mentioned earlier, previous studies showed that Group I alkenone signatures occur in a wide range of environmental conditions.

Comparison to Etang des Vallees, reported to have high concentrations of Group “EV” in the water column (Simon et al., 2013), showed that C_37:3a_Me and C_38a_Et were present in higher fractional abundance relative to the rest of the dataset (Supporting Information, Figure S3). However, this lake could experience warmer lake temperatures because it is shallow and experiences a mean annual air temperature of 10.4°C, which could also drive increases in the fractional abundance of longer chain alkenones. Therefore, it is difficult to draw any direct conclusions about the presence/absence of oligotypes and changes in alkenone abundance from our study, but this should be a focus of future efforts.

## 4 CONCLUSIONS

Samples from Alaska, Iceland, and Germany show that Group I haptophytes are more diverse and widespread than previously reported. By sequencing partial SSU rRNA genes, we identified a well‐supported clade within Group I, which includes samples from previously described Group “EV” (Simon et al., 2013). This clade is distinctly different from the “Greenland phylotype” and was present in almost all of the samples analyzed in this study. We also report the first LSU rRNA gene sequence‐based phylogeny for Group I Isochrysidales and the presence of distinct subclades; however, additional work is needed to better resolve these relationships and link them to SSU datasets. Using Lake E1 from Alaska as a case study, we noted a decrease in the relative read abundance of Group “EV” in the month of June 2016, and a corresponding increase in the “Greenland phylotype.” Despite the high genetic diversity in Group I Isochrysidales, we see an overall conservation of alkenones. In addition, we see a closer genetic relationship between Group I and III Isochrysidales.

Future work is needed to assess what controls the dominance of different oligotypes in lakes that are predominantly Group I. For example, when do Group “EV” Isochrysidales bloom relative to the “Greenland phylotype”? In addition, more work is needed to understand why Group I alkenones appear to be conserved across a chemically diverse set of lacustrine environments, and what role they play in the Isochrysidales life cycle.
