# Supplementary figures and images for: Phylogenetic diversity in freshwater‐dwelling Isochrysidales haptophytes with implications for alkenone production
Source: Geobiology. 2019 Feb 5;17(3):272–80. doi: 10.1111/gbi.12330 (PMC6590312; doi:10.1111/gbi.12330)

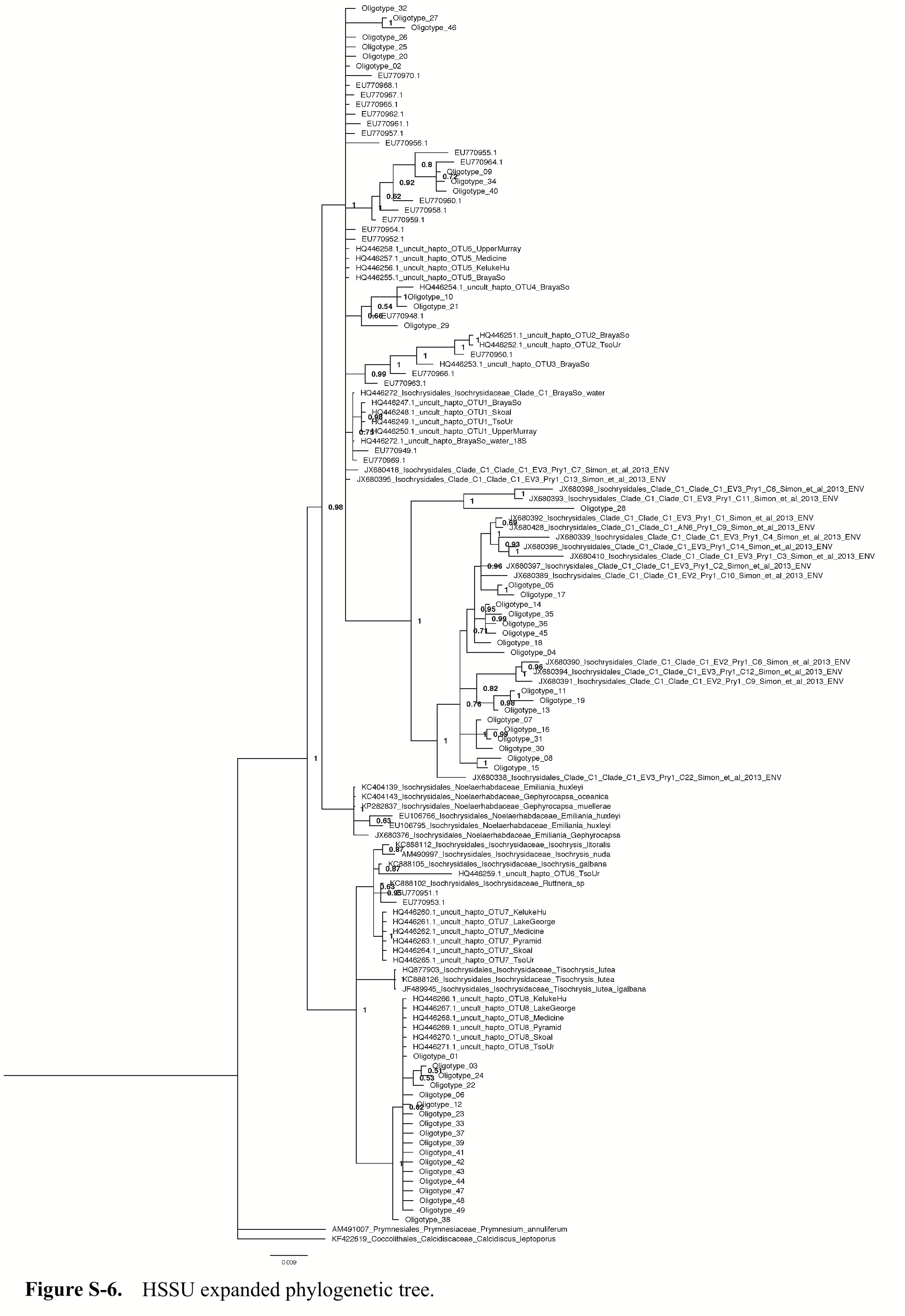

Supplement: Supplementary file 1 [file GBI-17-272-s001.tif]

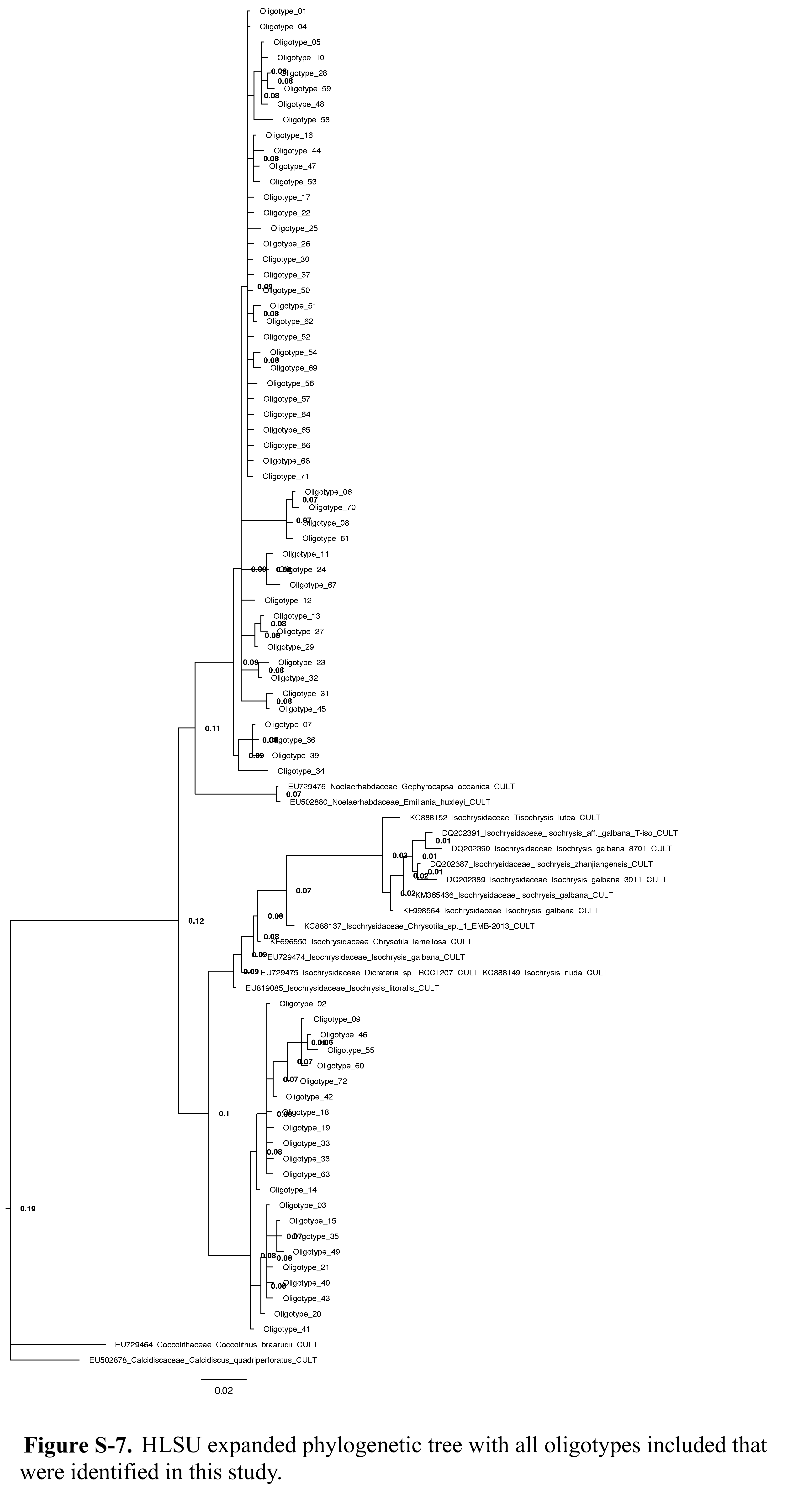

Supplement: Supplementary file 2 [file GBI-17-272-s002.tif]
