# Supplementary material for: Phylogenetic diversity in freshwater‐dwelling Isochrysidales haptophytes with implications for alkenone production
Source: Geobiology. 2019 Feb 5;17(3):272–80. doi: 10.1111/gbi.12330 (PMC6590312; doi:10.1111/gbi.12330)
Supplement: Supplementary file 4 [file GBI-17-272-s004.docx]

**Supporting information for:**

**Phylogenetic diversity in freshwater-dwelling Isochrysidales haptophytes with implications for alkenone production**

METHODS

*Study Sites & Sampling*

We selected study sites containing the Group I signature (Longo et al., 2016; 2018) from lakes distributed throughout the Northern Hemisphere. These sites included lakes in the U.S. (Alaska), Iceland, and Germany (see Figure S-1 and Table S-1a, b). In addition, we analyzed surface sediments from brackish Lake Fryxell, Antarctica as an external control due to good preservation of DNA and the presence of Group II Isochrysidales alkenone signatures (sediment sample from Rachael Morgan-Kiss). Prior to this study, Group II was shown to be the most closely related to Group I haptophytes (Edvardsen, Egge, & Vaulot, 2016), thus we selected a site where Group II was previously sequenced and alkenone signatures were studied. In addition, we wanted to contrast the diversity of Group I Isochrysidales with Group II and Group III with respect to both small subunit (SSU) and large subunit (LSU) ribosomal RNA (rRNA) sequences. However, taxon sampling for LSU is limited (Gran-Stadniczeñko et al., 2017), although it provides better branching support for certain parts of the tree. Additional sequences for Group II LSU sequences were generated in this study, alongside Group I, to address this issue.

We collected lake surface sediment and suspended particulate matter samples on the North Slope of Alaska in June 2016 from five lakes containing Group I alkenone signatures: E1, E5, Fog2, S6, and Toolik (Table S-1a, b). Unfortunately, some of our alkenone samples were compromised, and we are only presenting the data we were able to analyze. These lakes were selected for the wide range of environmental and chemical characteristics (e.g., pH, lake depth, water chemistry) and relatively high alkenone concentrations (Longo et al. 2016). We focused our sampling efforts around the spring transitional season, which was previously established as a period of heightened alkenone production in Alaska (Longo et al., 2016). We used a Van Dorn sampler to collect water samples at various depths, and a Hach^TM^ Hydrolab water column sensor array (temperature, pH, conductivity, dissolved oxygen content, and chlorophyll fluorescence) for concurrent water column measurements. We collected water samples for alkenones (4 L) and kept them in the cold and dark (for up to 2 hours) until the samples were vacuum filtered using combusted (450°C, 5 hrs) Whatman GF/F filters (0.7 µm). The filters were immediately frozen until analysis. Similarly, water samples (1 L from the same water column samples used for alkenones) were collected for DNA extraction, and remained in the dark and cold until we filtered them through Sterivex filters (0.22 µm; Millipore, Bedford, MA, USA), and immediately placed them at –20°C. Filters with 0.22µm pore size were used because Group I Isochrysidales were previously detected in the 0.22µm-5µm size fraction (Simon, López-García, Moreira, & Jardillier, 2013) and to ensure comparability with previous studies (e.g, D’Andrea et al., 2006; Theroux, Toney, Amaral-Zettler, & Huang, 2010). We took surface sediments with a gravity corer and subsampled the top 1 cm for alkenones and DNA. We collected all of our samples in Whirl-Pak^TM^ bags and immediately froze them (–20°C) and kept them in the dark until analysis.

Surface sediments from Germany were collected from the top 0-1 cm or 0-2 cm via sediment cores and were then freeze-dried and shipped to Brown University for processing as described by Longo et al. (2018). Sediment core samples from Iceland were obtained from the University of Minnesota National Lacustrine Core Facility (LacCore), Minneapolis, Minnesota, USA and shipped to Brown University for processing as described by Longo et al. (2018).

*Lipid analyses*

We freeze-dried and extracted the samples using established methods (e.g., Longo et al., 2016, 2018). In brief, we freeze-dried all of the filters and extracted them via sonication with dichloromethane:methanol (9:1 v/v), while we freeze-dried and extracted the surface sediments with a Dionex^TM^ accelerated solvent extraction (ASE) system at 120°C and 1200 psi. We separated all of the extracts by flash chromatography with silica gel (40-63 µm, 60 Å) columns to obtain alkane, ketone, and polar fractions. We then saponified the ketone fractions and purified them by silica gel column chromatography prior to analyzing them using gas chromatography-flame ionization detection (GC-FID). Alkenone concentrations were then quantified using an internal standard, 18-pentatriacontanone.

*DNA Extraction & Analysis*

We kept all of the samples either freeze-dried or frozen prior to analysis. The sediment samples (0.25 g) were then extracted using the MoBio PowerSoil^®^ DNA Isolation Kit (Qiagen, Carlsbad, CA, USA) according to the manufacturer’s instructions. We extracted all of the Sterivex filters using the Puregene Tissue Kit (Qiagen, Carlsbad, CA, USA) protocol. This was followed by quantification of DNA yields using a NanoDrop nucleic acid spectrophotometer (Thermo Scientific, Wilmington, DE). If needed, we performed additional clean-up using a DNeasy PowerClean Pro Kit (Qiagen, Carlsbad, CA, USA). Samples were then amplified using: (1) haptophyte-specific primers targeting the V4 hypervariable region of the small-subunit rRNA gene (hereon designated HSSU; Egge et al., 2013); (2) haptophyte-specific primers targeting the D1/D2 hypervariable region of the large-subunit rRNA gene (hereon designated HLSU; Bittner et al., 2013); and, (3) eukaryotic-specific primers targeting the V4 hypervariable region of the SSU rRNA gene in eukaryotes (hereon designated Ev4; Balzano, Abs, & Leterme, 2015; Stoeck et al., 2010; see Table S-2).

For Ev4, we used fusion primers for PCR using the conserved regions of the Eukaryotic V4 sequence based on Balzano et al., (2015) and Stoeck et al., (2010) (see Table S-2). Illumina adaptors, consisting of 24 unique inline barcodes and 8 indices, enabled us to sequence 192 samples with unique barcode-index combinations in a single Illumina MiSeq run. We carried out all polymerase chain reactions (PCRs) in triplicate with 33 µL using a combination of 1.0 U Phusion High-Fidelity DNA Polymerase (New England BioLabs, Ipswich, MA) with x1 Phusion High-Fidelity buffer, 200 µM dNTP mix, 1.5 mM MgCl_2_, DMSO, and 0.2 µM of each primer. Approximately, 5-20 ng of template DNA was added to each PCR and a no-template control was run. We conducted PCR reactions using an Eppendorf Gradient Thermocycler (Hamburg, Germany) with the following PCR conditions for Ev4: initial denaturing for 30 s at 98°C, denaturing for 10 s at 98°C, followed by 30 s of annealing at 53°C, primer extension for 30 s at 72°C, repetition from step 2 for 10 cycles, followed by 98°C for 10 s, 48°C for 30 s, 72°C for 30 s with 15 cycles, and a final extension for 2 min at 72°C. We verified the resulting PCR products using the Caliper LabChip GX (Caliper Life Sciences, Hopkinton, MA). The amplicons were cleaned with Agencourt AMPure XP magnetic beads (Agencourt Bioscience Corporation/Beckman Coulter, Beverly, MA) and quantified with Quant-iT PicoGreen dsDNA Assay Kit (Molecular Probes, Inc., Eugene, OR). The amplicons were equimolarly pooled and we verified the size using the Agilent Bioanalyzer DNA 1000 chip (Agilent Technologies, Santa Clara, CA). The concentration of the amplicon pool was further analyzed with qPCR prior to sequencing on an Illumina MiSeq (Illumina, Inc., San Diego, CA) 500-cycle paired-end run at the Marine Biological Laboratory W. M. Keck Ecological and Evolutionary Genetics Facility.

Fusion primer sequences consisted of 528F_long_HAP_454 and PRYM01+7_HAP454 for HSSU (Table S-2) and HAP_LSU_F and LHapto20R_bis for HLSU (Table S-2). Similarly, Illumina-specific adaptors, with 6 unique inline barcodes and 8 indices, enabled us to distinguish and sequence 48 samples for HSSU and HLSU. We carried out triplicate PCR reactions for each genomic DNA sample with a 33 µL cocktail of 1.0 U Platinum High-Fidelity DNA Polymerase (Life Technologies, Carlsbad, CA): with x1 Phusion High-Fidelity buffer, 200 µM dNTP mix, 1.5 mM MgSO_4_, and 0.2 µM of each primer. We added 5-20 ng of template DNA to each PCR and included a no-template control for the run. The PCR conditions for HSSU was as follows: initial denaturing for 30 s at 94°C, denaturing for 15 s at 94°C, followed by 30 s of annealing at 55°C, primer extension for 60 s at 68°C, with repetition at step 2 for 30 cycles, and final extension for 5 min at 68°C. We used the following HLSU PCR conditions: initial denaturing for 2 min at 94°C, denaturing for 30 s at 94°C, followed by 30 s of annealing at 55°C, primer extension for 3 min at 68°C, with repetition from step 2 for 35 cycles, and final extension for 5 min at 68°C. The resulting PCR triplicate reactions were pooled and the PCR products were verified using a Caliper LabChip GX. We cleaned the amplicons with Agencourt AMPure XP magnetic beads and quantified the amplicons with Quant-iT PicoGreen dsDNA Assay Kit. The amplicons were equimolarly pooled and the size was verified using the Agilent Bioanalyzer DNA 1000 chip. In addition, we checked the concentration of the amplicon pool with qPCR prior to sequencing on an Illumina MiSeq 500-cycle paired-end run. The pool of amplicon libraries was diluted to 13 pM prior to clustering while PhiX was added as a diversity control for the MiSeq sequencing run. All sequences are available through Visualization and Analysis of Microbial Population Structures (https://vamps2.mbl.edu/) and were deposited in the National Center for Biotechnology Information (NCBI) Sequence Read Archive (SRA) with the following numbers: SAMN09475369 - SAMN09475400 for HSSU sequences and SAMN09475416 - SAMN09475436 for HLSU sequences. A minimum information about a marker gene sequence (MIMARKS) table is included in the supplements (see supporting information, Table S-3).

*Bioinformatics*

All sequences were demultiplexed using the unique barcodes, and then merged for quality control. Unique sequences were then identified and checked for chimeras. The initial taxonomy was assigned using a Global Alignment for Sequence Taxonomy (GAST) pipeline (Huse et al., 2008). A search using SILVA 119 taxonomy (Pruesse et al., 2007; Quast et al., 2013) did not produce reasonable hits for either HSSU or HLSU, thus a new reference database was created using SSU and LSU sequences from Edvardsen et al. (2016). The results were checked with the NCBI Basic Local Alignment Tool (NCBI BLAST; Altschul et al., 1997). All Isochyrsidales sequences were subsequently uploaded into ARB (Ludwig et al., 2004) for initial comparison with the existing SILVA 119 database (Pruesse et al., 2007; Quast et al., 2013). Overlap between the HSSU and Ev4 sequences (minimum 340 bp) was used to align the sequences to reference SSU rRNA sequences from Edvardsen et al., (2016) in PyNAST (Caporaso et al., 2010), and then checked manually. Similarly, HLSU sequences were aligned to LSU reference sequences from Edvardsen et al., (2016) in PyNAST (Caporaso et al., 2010) and checked manually. We employed entropy as implemented in Oligotyping to analyze the resulting reads and generate Exact Sequence Variants ("oligotypes"; Callahan, McMurdie, & Holmes, 2017; Eren et al., 2013, 2014), which are independent of the reference database and will allow for comparability in future studies. For our analysis, we required that each oligotype occur at least 100 times in one or more sample to be considered a valid oligotype, this was based on the average number of reads per sample and was used to reduce noise from oligotypes with nominal abundance (Eren et al. 2013). A conservative estimate of 100 was used. The resulting oligotypes were then combined into one file with all available Group I Isochrysidales sequences (D’Andrea et al., 2006; Edvardsen et al., 2016; Gran-Stadniczeñko et al., 2017; Theroux et al., 2010) and compared against previously defined OTUs by Theroux et al. (2010) and D’Andrea et al. (2006) to check for overlap. PAUP* (v4.0b10; Swofford, 2003) and ModelTest (3.06; Posada & Crandall, 1998) were used to select a substitution model that best described the DNA data. For both HSSU (340 bp) and HLSU (370 bp) a general time reversible (GTR) model with gamma substitution rates was chosen. These models were used to infer phylogenetic trees in MrBayes (v3.2.6; Ronquist et al., 2012) for HSSU and HLSU. All phylogenetic trees were inferred using two simultaneous runs with 3 heated chains (chain temperature = 0.1) and one cold chain that consisted of a 25% burn in frequency and sampling frequency of 100. Analyses were stopped when the standard deviations of the split frequencies reached values below 0.01, such that the HSSU tree was run for 3.0 $\times$ 10^6^ generations, and both the HLSU tree with all oligotypes and the simplified HLSU tree were run for 3.0 $\times$ 10^6^ generations. The resulting trees were visualized in FigTree (v1.4.3; Rambaut, 2007).

*Alkenone proxy calculations*

To test for concurrent changes in alkenones and Group I haptophytes, we extracted and measured the fractional abundances and concentrations of 16 alkenones in our suite of Alaskan lakes (see supporting information, Table S-4). The ratio of isomeric ketones, RIK_37_ index, was determined using changes in fractional abundance of C_37:3a_Me compared to C_37:3b_Me alkenones to test for Group I and II mixing (Longo et al., 2016):

$\text{RIK}_{\text{37}}\text{= }\frac{\text{C}_{\text{37:3a}}\text{Me}}{\text{C}_{\text{37:3a}}\text{Me+ }\text{C}_{\text{37:3b}}\text{Me}}$ (1)

A RIK_37_ value of 1.0 indicates that only C_37:3a_Me is present, suggesting alkenone production by Group II, and values from 0.48-0.63 indicates the presence of both C_37:3_Me ketone isomers and alkenone production by Group I (Longo et al., 2018). Temperature calibrations are commonly determined using the $\text{U}_{\text{37}}^{\text{K}}$ index (Brassell et al., 1986), which was later modified and calibrated for sea surface temperatures (Müller, Kirst, Ruhland, von Storch, & Rosell-Melé, 1998; Prahl & Wakeham, 1987) and further modified (Longo et al. 2016) to incorporate the C_37:3b_Me isomer for lacustrine calibrations.

$\text{U}_{\text{37}}^{\text{K}}\text{= }\frac{\text{C}_{\text{37:2}}\text{Me - }\text{C}_{\text{37:4}}\text{Me}}{{\text{C}_{\text{37:2}}\text{Me + C}}_{\text{37:3a}}\text{Me + }\text{C}_{\text{37:3b}}\text{Me + }\text{C}_{\text{37:4}}\text{Me}}$ (2)

For Lake E1, Alaska we regressed calculated $\text{U}_{\text{37}}^{\text{K}}$ index values against *in situ* water column temperatures to assess the similarity between Group I temperature responses in neighboring, but physically and chemically distinct lakes. To assess whether differences in Group I diversity corresponded with significant variations in alkenones, we compiled previously published alkenone data (see Table S-4; Longo et al., 2016; 2018) for comparison with DNA analyses from this study. We expressed alkenone quantifications as fractional abundances, in which each alkenone concentration is divided by the summed concentration data for all 16 alkenones in a given sample. The resulting data was graphically visualized using a heatmap (Figure S-3) with the ggplot2 package (Wickham, 2009) in R (v. 3.4.1).

REFERENCES

Altschul, S.F., Madden, T.L., Schäffer, A.A., Zhang, J., Zhang, Z., Miller, W., & Lipman, D.J. (1997). Gapped BLAST and PSI-BLAST: a new generation of protein database search programs. *Nucleic Acids Research*, 25, 3389–3402. https://doi.org/10.1093/nar/25.17.3389

Balzano, S., Abs, E., & Leterme, S.C. (2015). Protist diversity along a salinity gradient in a coastal lagoon. *Aquatic Microbial Ecology*, 74, 263–277. https://doi.org/10.3354/ame01740

Bittner, L., Gobet, A., Audic, S., Romac, S., Egge, E.S., Santini, S., Ogata, H., Probert, I., Edvardsen, B., & de Vargas, C. (2013). Diversity patterns of uncultured Haptophytes unravelled by pyrosequencing in Naples Bay. *Molecular Ecology*, 22, 87–101. https://doi.org/10.1111/mec.12108

Brassell, S.C., Eglinton, G., Marlowe, I.T., Pflaumann, U., & Sarnthein, M. (1986). Molecular Stratigraphy: a new tool for climatic assessment. *Nature*, 320, 129-133. https://doi.org/10.1038/320129a0

Callahan, B.J., McMurdie, P.J., & Holmes, S.P. (2017). Exact sequence variants should replace operational taxonomic units in marker-gene data analysis. *The ISME Journal*, 11, 2639–2643. https://doi.org/10.1038/ismej.2017.119

Caporaso, J.G., Bittinger, K., Bushman, F.D., DeSantis, T.Z., Andersen, G.L., & Knight, R. (2010). PyNAST: A flexible tool for aligning sequences to a template alignment. *Bioinformatics*, 26, 266–267. https://doi.org/10.1093/bioinformatics/btp636

D’Andrea, W.J., Lage, M., Martiny, J.B.H., Laatsch, A.D., Amaral-Zettler, L.A., Sogin, M.L., & Huang, Y. (2006). Alkenone producers inferred from well-preserved 18S rDNA in Greenland lake sediments. *Journal of Geophysical Research*, 111. https://doi.org/10.1029/2005JG000121

Edvardsen, B., Egge, E.S., & Vaulot, D. (2016). Diversity and distribution of haptophytes revealed by environmental sequencing and metabarcoding – a review. *Perspectives in Phycology*, 3, 77–91. https://doi.org/10.1127/pip/2016/0052

Egge, E., Bittner, L., Andersen, T., Audic, S., de Vargas, C., & Edvardsen, B. (2013). 454 pyrosequencing to describe microbial eukaryotic community composition, diversity and relative abundance: a test for marine haptophytes. *PLoS One*, 8 (9). https://doi.org/10.1371/journal.pone.0074371

Eren, A.M., Maignien, L., Sul, W.J., Murphy, L.G., Grim, S.L., Morrison, H.G., & Sogin, M.L. (2013). Oligotyping: Differentiating between closely related microbial taxa using 16S rRNA gene data. *Methods in Ecology and Evolution*, 4, 1111–1119. https://doi.org/10.1111/2041-210X.12114

Eren, A.M., Borisy, G.G., Huse, S.M., & Mark Welch, J.L. (2014). Oligotyping analysis of the human oral microbiome. *Proceedings of the National Academy of Sciences,* 111, E2875–E2884. https://doi.org/10.1073/pnas.1409644111

Huse, S.M., Dethlefsen, L., Huber, J.A., Welch, D.M., Relman, D.A., & Sogin, M.L. (2008). Exploring microbial diversity and taxonomy using SSU rRNA hypervariable tag sequencing. *PLoS Genetics*, 4. https://doi.org/10.1371/journal.pgen.1000255

Longo, W.M., Theroux, S., Giblin, A.E., Zheng, Y., Dillon, J.T., & Huang, Y. (2016). Temperature calibration and phylogenetically distinct distributions for freshwater alkenones: Evidence from northern Alaskan lakes. *Geochimica et Cosmochimica Acta*, 180, 177–196. https://doi.org/10.1016/j.gca.2016.02.019

Longo, W.M., Huang, Y., Yao, Y., Zhao, J., Giblin, A.E., Wang, X., … Shinozuka, Y. (2018). Widespread occurrence of distinct alkenones from Group I haptophytes in freshwater lakes: Implications for paleotemperature and paleoenvironmental reconstructions. *Earth and Planetary Science Letters*, 492, 239–250. https://doi.org/10.1016/j.epsl.2018.04.002

Ludwig, W., Strunk, O., Westram, R., Richter, L., Meier, H., Yadhukumar, A., … Schleifer, K.H. (2004). ARB: A software environment for sequence data. *Nucleic Acids Research*, 32, 1363–1371. https://doi.org/10.1093/nar/gkh293

Müller, P.J., Kirst, G., Ruhland, G., von Storch, I., & Rosell-Melé, A. (1998). Calibration of the alkenone paleotemperature index $U_{37}^{K'}$ based on core-tops from the eastern South Atlantic and the global ocean (60°N-60°S). *Geochimica et Cosmochimica Acta*, 62, 1757–1772. https://doi.org/10.1016/S0016-7037(98)00097-0

Posada, D., & Crandall, K.A. (1998). MODELTEST: Testing the model of DNA substitution. *Bioinformatics (Oxford, England)*, 14, 817–818. https://doi.org/10.1093/bioinformatics/14.9.817

Prahl, F.G., & Wakeham, S.G. (1987). Calibration of unsaturation patterns in long-chain ketone compositions for palaeotemperature assessment. *Nature*, 330, 367–369. https://doi.org/10.1038/330367a0

Pruesse, E., Quast, C., Knittel, K., Fuchs, B.M., Ludwig, W., Peplies, J., & Glöckner, F.O. (2007). SILVA: A comprehensive online resource for quality checked and aligned ribosomal RNA sequence data compatible with ARB. *Nucleic Acids Research*, 35, 7188–7196. https://doi.org/10.1093/nar/gkm864

Quast, C., Pruesse, E., Yilmaz, P., Gerken, J., Schweer, T., Yarza, …Glöckner, F.O. (2013). The SILVA ribosomal RNA gene database project: Improved data processing and web-based tools. *Nucleic Acids Research*, 41, D590–D596. https://doi.org/10.1093/nar/gks1219

Rambaut, A., (2007). Molecular evolution, phylogenetics and epidemiology: FigTree [Software]. Available from http://tree.bio.ed.ac.uk/software/figtree/ (accessed 7.3.18).

Ronquist, F., Teslenko, M., Van Der Mark, P., Ayres, D.L., Darling, A., Höhna, S., …Huelsenbeck, J.P. (2012). MrBayes 3.2: efficient Bayesian phylogenetic inference and model choice across a large model space. *Systematic Biology*, 61, 539–542. https://doi.org/10.1093/sysbio/sys029

Simon, M., López-García, P., Moreira, D., & Jardillier, L. (2013). New haptophyte lineages and multiple independent colonizations of freshwater ecosystems. *Environmental Microbiology Reports*, 5, 322–332. https://doi.org/10.1111/1758-2229.12023

Stoeck, T., Bass, D., Nebel, M., Christen, R., Jones, M.D.M., Breiner, H.W., & Richards, T.A. (2010). Multiple marker parallel tag environmental DNA sequencing reveals a highly complex eukaryotic community in marine anoxic water. *Molecular Ecology*, 19, 21–31. https://doi.org/10.1111/j.1365-294X.2009.04480.x

Swofford, D.L. (2003). PAUP*: Phylogenetic analysis using parsimony, version 4.0 b10.

Theroux, S., D'Andrea, W.J., Toney, J., Amaral-Zettler, L., & Huang, Y. (2010). Phylogenetic diversity and evolutionary relatedness of alkenone-producing haptophyte algae in lakes: Implications for continental paleotemperature reconstructions. *Earth and Planetary Science Letters*, 300, 311–320. https://doi.org/10.1016/j.epsl.2010.10.009

Wickham, H. (2009). ggplot2: Elegant Graphics for Data Analysis. Springer-Verlag New York.
