# Supplementary material for: Phylogenetic diversity in freshwater‐dwelling Isochrysidales haptophytes with implications for alkenone production
Source: Geobiology. 2019 Feb 5;17(3):272–80. doi: 10.1111/gbi.12330 (PMC6590312; doi:10.1111/gbi.12330)
Supplement: Supplementary file 5 [file GBI-17-272-s005.pdf]

Supporting information for:

Phylogenetic diversity in freshwater-dwelling Isochrysidales haptophytes with implications for alkenone production

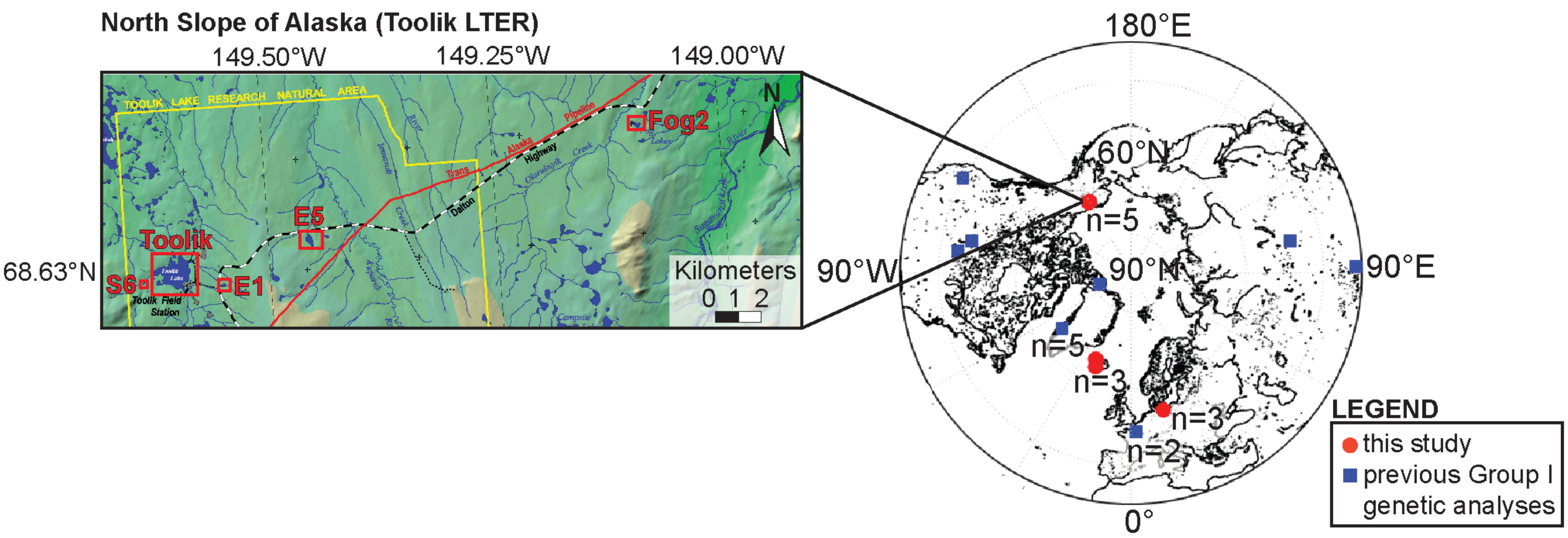

Figure S-1. The map on the left, is a map of the Northern Hemisphere with samples used in this study indicated by red circles, and blue squares marking locations where previous genetic analyses confirmed the presence of Group I Isochrysidales. Markers with an "n" indicate the number of lakes represented at each location. On the right, there is a zoomed in map of the North Slope of Alaska of the area in and around the Toolik Long Term Ecological Research (LTER) station with the five lakes used in our case study indicated in red: E1, E5, Fog2, S6, and Toolik (this image was modified from a figure by the Institute of Arctic Biology, 2012).

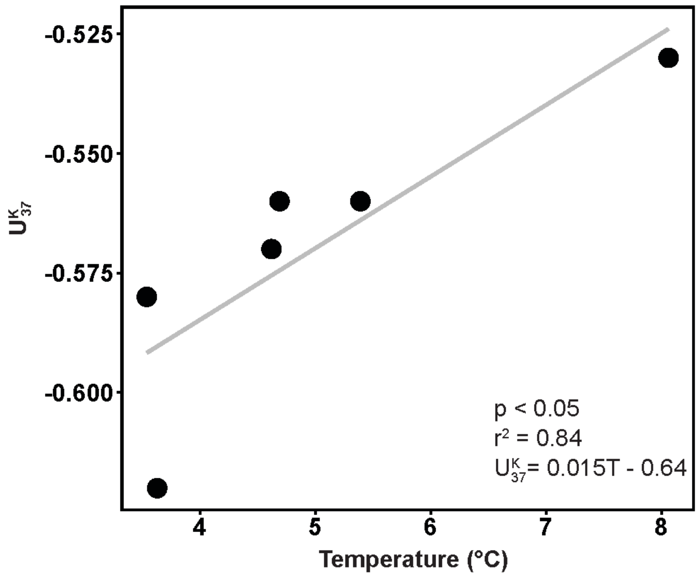

Figure S-2. A comparison of U^K_{37} index with water column temperatures from Lake E1 at both 3- and 10-m depth from samples taken in June 2016.

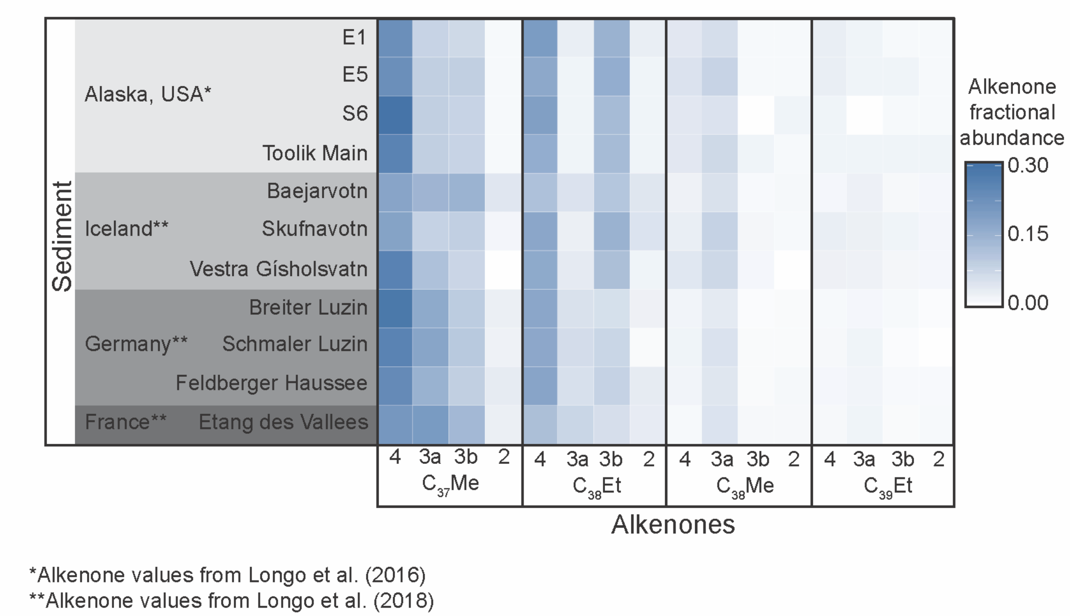

Figure S-3. Comparison of alkenone fractional abundance compiled from previous studies (Longo et al. 2016, 2018) for samples analyzed and discussed in this study.

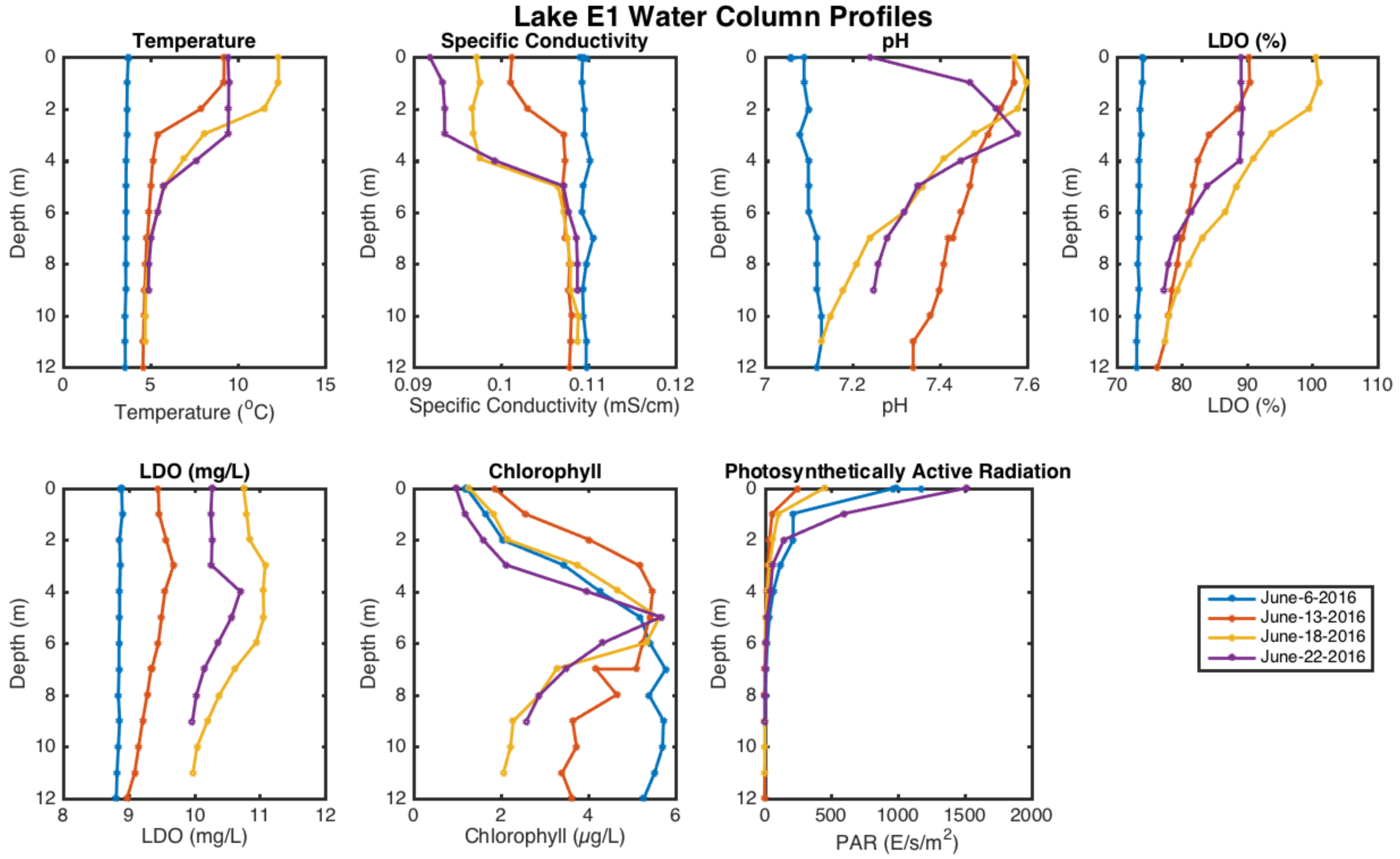

Figure S-4. Water column profiles for Lake E1, Alaska from June 2016.

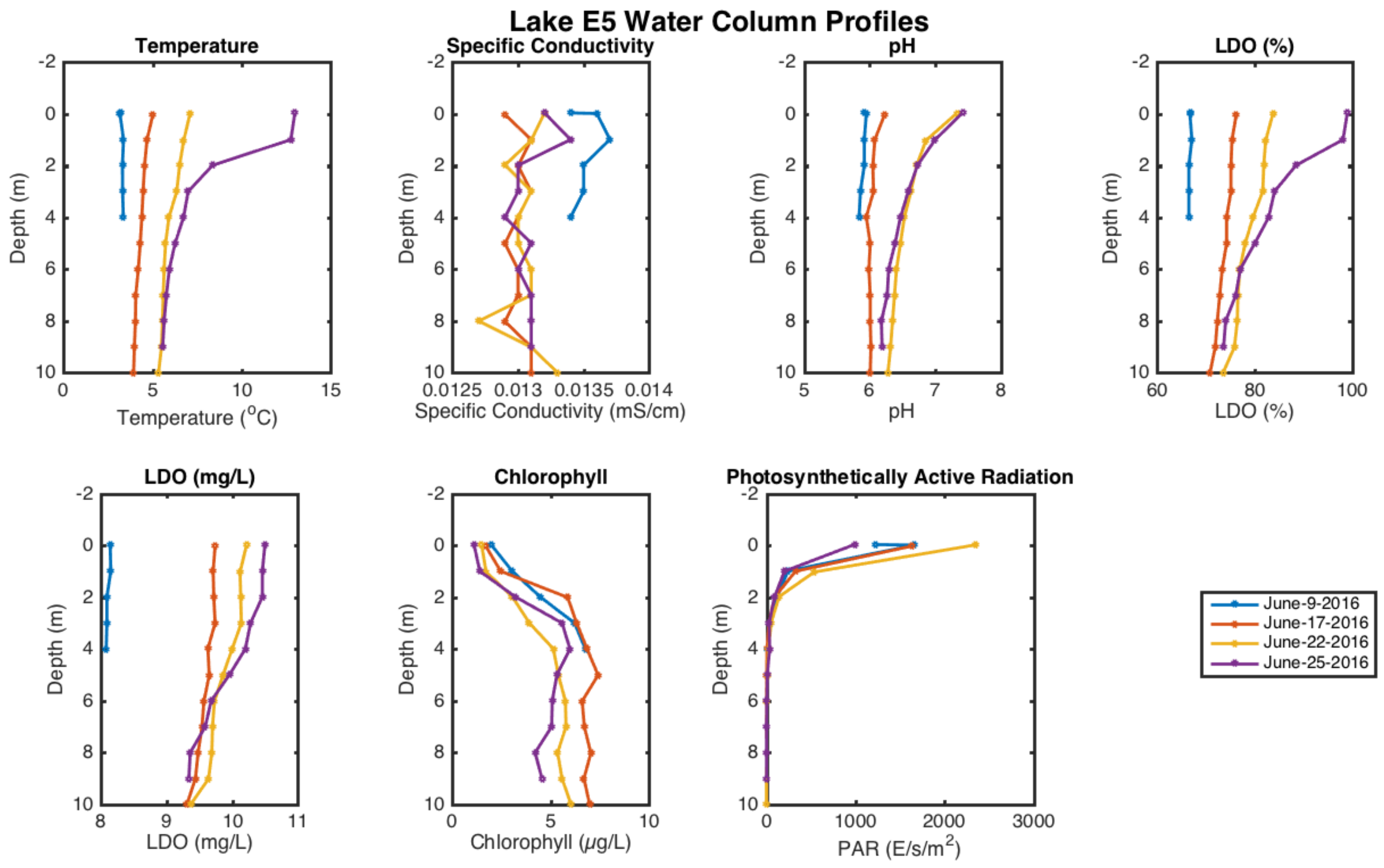

Figure S-5. Water column profiles for Lake E5, Alaska from June 2016.

Figure S-6. HSSU expanded phylogenetic tree *(see Figure S-6)*.
Figure S-7. HLSU expanded phylogenetic tree with all oligotypes included that were identified in this study *(see Figure S-7)*.

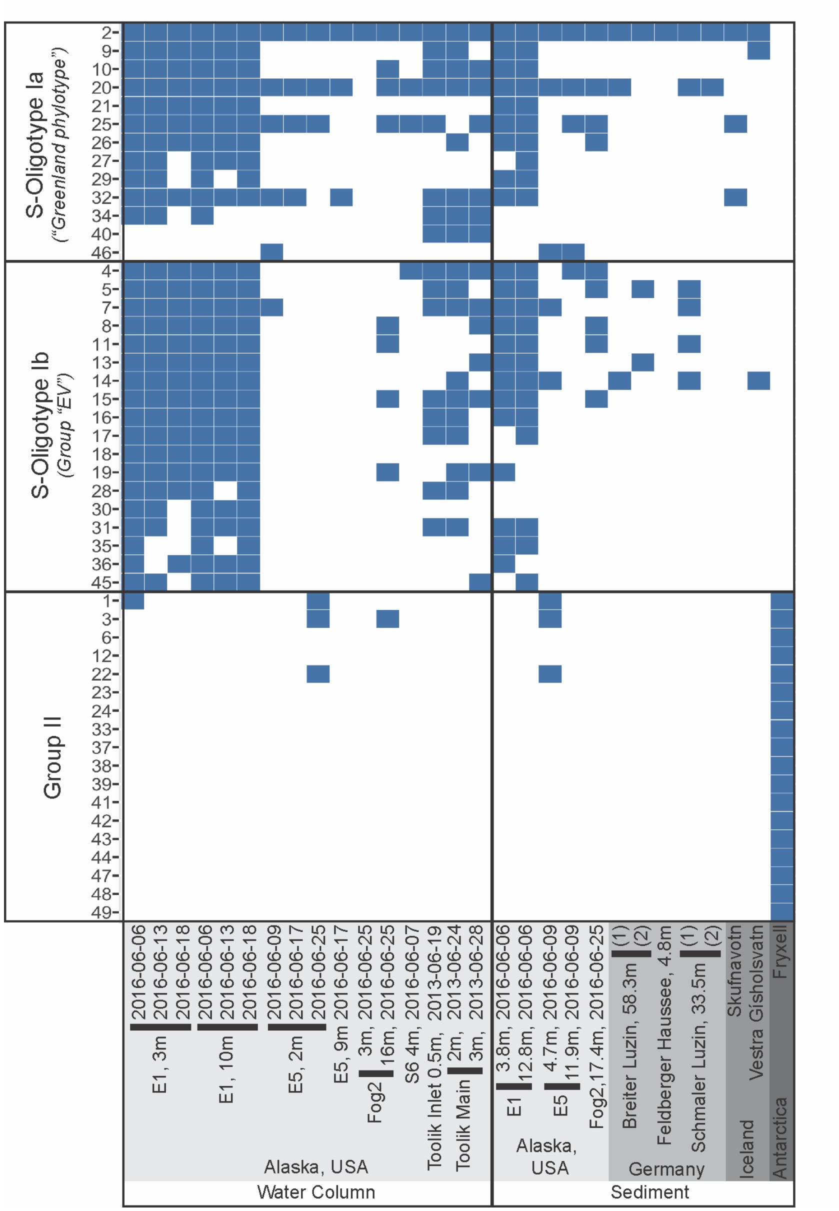

Figure S-8. A binary heatmap, indicating the presence/absence of HSSU oligotypes from our samples where a blue square corresponds to the presence of a certain oligotype and a white space indicates the absence of a specific oligotype. The y-axis corresponds to the oligotypes identified (e.g., Oligotype 1) and the x-axis indicates the sampling location.

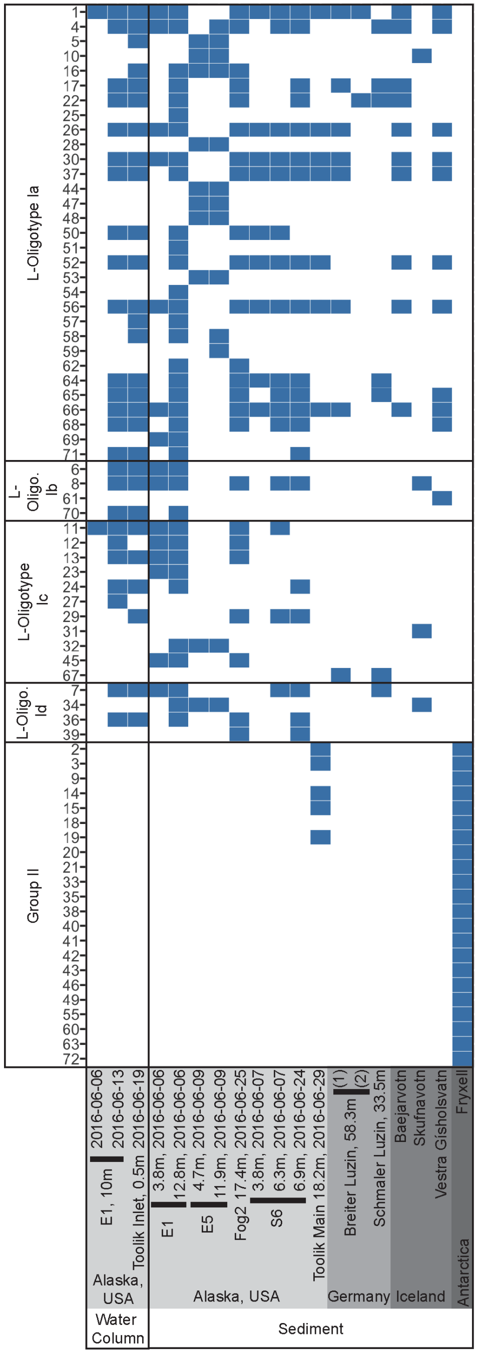

Figure S-9. A binary heatmap, indicating the presence/absence of HLSU oligotypes from our samples where a blue square corresponds to the presence of a certain oligotype and a white space indicates the absence of a specific oligotype. The y-axis corresponds to the oligotypes identified (e.g., Oligotype 1) and the x-axis indicates the sampling location.

Supplemental Tables.

Table S-1a. Water column samples from Alaskan lakes.

Location			Sample Information			Environmental Parameters		
Lake	Latitude	Longitude	Date Sampled	Sample Depth (m)	Depth at Sampling Location (m)	pH	Water Temperature (°C)	Lake Physical Status
E1	68.63	-149.55	6-Jun-2016	3	12.82	7.08	3.63	Ice-covered
			6-Jun-2016	10	12.82	7.13	3.54	Ice-covered
			13-Jun-2016	3	12.43	7.51	5.39	Mixing
			13-Jun-2016	10	12.43	7.38	4.62	Mixing
			18-Jun-2016	3	11.93	7.48	8.06	Summer stratification
			18-Jun-2016	10	11.93	7.15	4.69	Summer stratification
E5	68.64	-149.46	9-Jun-2016	2	4.86	5.92	3.17	Mixing
			17-Jun-2016	2	10.12	6.05	4.55	Mixing
			17-Jun-2016	9	10.12	6.02	3.98	Mixing
			25-Jun-2016	2	9.5	6.73	8.37	Summer stratification
Fog2	68.679	-149.09	25-Jun-2016	3	16.59	7.22	5.72	Summer stratification
			25-Jun-2016	16	16.59	7.17	3.49	Summer stratification
S6	68.63	-149.64	7-Jun-2016	4	6.25	-	-	Mixing
Toolik Inlet	68.63	-149.6	19-Jun-2013	0.5	-	-	-	Summer stratification
Toolik Main	68.63	-149.6	24-Jun-2013	2	25	-	-	Summer stratification
			28-Jun-2013	3	25	-	-	Summer stratification

Table S-1b. Sediment samples from Alaskan and global lakes.

Location				Sample Information			Environmental Parameters				
Lake	Country	Latitude	Longitude	Date Sampled	Sample Type	Lake Sampling Depth (m)	Maximum Lake Depth (m)	pH	Salinity (ppt)	Mean Spring Temperature (°C)	Data Source
E1	USA	68.63	-149.55	6-Jun-2016	Surface Sediment	3.8	13	7.76	0	-	Longo et al. 2016, 2018
				6-Jun-2016	Surface Sediment	12.8					
E5	USA	68.64	-149.46	9-Jun-2016	Surface Sediment	4.7	13	6.84	0	-0.25	Longo et al. 2016, 2018
				17-Jun-2016	Surface Sediment	11.9					
Fog2	USA	68.679	-149.09	25-Jun-2016	Surface Sediment	17.4	20	8.04	0	-	Longo et al. 2016, 2018
S6	USA	68.63	-149.64	7-Jun-2016	Surface Sediment	3.8	9	8.48	0	-0.025	Longo et al. 2016, 2018
				7-Jun-2016	Surface Sediment	6.3					
				24-Jun-2016	Surface Sediment	6.9					
Toolik Main	USA	68.63	-149.6	29-Jun-2016	Surface Sediment	18.2	26	7.61	0	-	Longo et al. 2016, 2018
Breiter Luzin	Germany	53.35	13.46	2016	Surface Sediment x1	58.3	58.3	8.70	0.2	2.05	Longo et al. 2018
				2016	Surface Sediment x2	58.3					
Schmaler Luzin	Germany	53.32	13.44	2016	Surface Sediment x1	33.5	33.5	8.50	-	2	Longo et al. 2018
				2016	Surface Sediment x2	33.5					
Felberger Haussee	Germany	53.35	13.45	2016	Surface Sediment	4.8	12.5	8.90	0.3	2.025	Longo et al. 2018
Baejarvotn	Iceland	65.73	-21.43	-	Core (8.5-10.5 cm)	23.2	23.2	7.40	0	-	Longo et al. 2018
Skufnavotn	Iceland	65.89	-22.12	9-Mar-2010	Core (19-21 cm)	4.5	5.8	-	-	-	Longo et al. 2018
Vestra Gísholtsvatn	Iceland	63.95	-20.52	4-Aug-2008	Core (5.5-7.5 cm)	13.4	13.4	7.70	-	-	Longo et al. 2018
Fryxell	Antarctica	-77.61	85.54	-	Surface Sediment	-	18	8.04	-	-	Wharton et al. 1982; Yang et al. 2015

Table S-2. Primers used in this study.

Primer Name	Target Group	Target Region	Direction 5' - 3'	Primer 5' - 3'	Length (bp)	References
V4F	Eukaryotes	18S-V4	Forward	CCAGCASCYGCGGTAATTCC	380	Stoeck et al. 2010
V4RB	Eukaryotes	18S-V4	Reverse	ACTTTCGTTCTTGATYRR	380	Balzano et al. 2015
528F_long	Haptophytes	18S	Forward	GCGGTAATTCCAGCTCCA	400	Egge et al. 2013
PRYM01+7	Haptophytes	18S	Reverse	GATCAGTGAAAACATCCCTGG	400	Egge et al. 2013
HAP_LSU_F	Haptophytes	28S	Forward	GGTRTCGGAGARGGTGAGAATCC	350-400	Modified from Bittner et al. 2013
LHapto20R_bis	Haptophytes	28S	Reverse	TCAGACTCCTTGGTCCGTGTTTCT	350-400	Bittner et al. 2013

(See SI-Tables.xlsx)
Table S-3. Minimum information about a marker gene seqeunce (MIMARKS) table for HSSU and HLSU sequences analyzed in this study. All sequences are available through Visualization and Analysis of Microbial Population Structures (https://vamps2.mbl.edu/) and were deposited in the National Center for Biotechnology Information (NCBI) Sequence Read Archive (SRA) with the following accession numbers: SAMN09475369 - SAMN09475400 for HSSU sequences and SAMN09475416 - SAMN09475436 for HLSU sequences. *(see tab Table S-3)*.
Table S-4. Alkenone fractional abundance data for the samples analyzed for alkenones in this study *(see tab Table S-4)*.
Table S-5. The number of sequence counts used to define each HSSU oligotype *(see tab Table S-5)*.
Table S-6. The number of sequence counts used to define each HLSU oligotype *(see tab Table S-5)*.

Table S-7. HSSU unique oligotypes showing the variable positions that were used to define each oligotype (note that this is not the entire sequence) with the number of sequences used to define each oligotype listed in the last column. The unique oligotype sequences are shown with nucleotide variations (relative to Oligotype 1) highlighted in blue to indicate what differences were used to define each oligotype.

HSSU Oligotypes	Unique Oligotypes	Sequences per Oligotype
Oligotype 1	GTGTTGCGTCGTCGCCCAGCCTGGTGCCTGTCCGTGAAATGTTGGGCTTCAGAAGTGCGGTCG	73998
Oligotype 2	GTGTTGCGTCGTCCGCCGTCCCGCGCCCGCCGCGCGAAATGTTGGGCTTCAAAAGTGCGGCCG	40771
Oligotype 3	GTGTTGCGTCGTCGCTCAGCCTGGTGCCTGTCCGTGAAATGTTGGGCTTCAGAAGTGCGGTCG	29167
Oligotype 4	A-GTAGCGTCATCTGCCGTCCCGCGCCCGCCGCCCAA-ATGTTGGGCTTCAAGAGTGTGGCTG	5248

Oligotype 5	A-GTTGCGTCGTCTCCCGTCCCGCGCCCGCCGCGCGAAAAGTTGCGCTTTAAGAGTGTGGCCG	4286
Oligotype 6	GTGTTGCGTCGTCGCCCAGCCTGGTGCCTGTCCGTGAAATGTTGGGCTTCAGAAGGGCGGTCG	3394
Oligotype 7	GTGTTGCGTCGTCTGCCGTCCCGCGCCCGCCGCGCGAAATGTTGGGCTCCAAGAGTATGGCCG	3366
Oligotype 8	GTGTTGCGTTGT-TGCCGTCCCGCGCCCGACGCGCGAAATGTTGGGCTTCAAGAGTGTGGCCG	3084
Oligotype 9	GCGTTGCGTCGTCCGCCGTCCCGCGCCCGCCGCGCGAAATGTTGGGCTTCAAAAATGCGGCCG	3021
Oligotype 10	GTGTTGCGTCGTCCGCCGTCCCGCGCCCGCCGCGCGAAATGTTGGGCTTCAAATATGCGACCG	3006
Oligotype 11	GTGTCGCTCCGTCTGCCGTCCCGCGCCCGCCGCGCGAAATGTTTGGCTTCAAGAGTGTGGCTG	2513
Oligotype 12	GTGTTGCGTCGTCGCCCAGCCTGGTGCCTGTCCGTGAAATGTTGGGCTTCAGAGGTGCGGTCG	2413
Oligotype 13	GTGTCGCGTCGTCTGCCGTCCCGCGCCCGCCGCGCGAAATGTTTGGCTTCAAGAGTGTGGCTG	2280
Oligotype 14	A-GTTGCGTCGTCTCCCGTCCCGCGCCCGCCGCGCGAAATGTTGGGCTTCTAGAGTGTGGCCG	2121
Oligotype 15	GTGTTGCGTCGT-TGCCGTCCTGCGCCCGACGCGCGAAATGTTGGGCTTCAAGAGTGTGGCCG	1930
Oligotype 16	GTGTTGCGTCGTCTGCCGTCCCGCGCCCGCCGCGCGTAATATTGGGCTCCAAGAGTATGGCCG	1899
Oligotype 17	A-GCTGCGTTGTCTCCCGTCCCGCGCCCGCCGCGCGAAAAGTTGCGCTTTAAGAGTGTGGCCG	1801
Oligotype 18	A-GTTGCGTCGTCTCCCGTCCCGCGCCCGACGCGCGAAATGTTGGGCTTCAAGAGTGTGGCCG	1765
Oligotype 19	GTGTCGCTCCGTCTGCCGTCCCGCGCCCGCCGCGTGAAATGTTTGGCTTCAAGAGTGTGGCTG	1393
Oligotype 20	GTGTTGCGTCGTCCGCCGTCCCGCGCCCGCCGCGCGAAATGTTGGGCTTCAAAGGTGCGGCCG	1331
Oligotype 21	GTGTTGCGTCGTCCGCCGTCCCGCGCACGCCGCGCGAAATGTTGGGC-TCAAATATGCGACCG	1288
Oligotype 22	GTGTTGCGTCGTCGCTCAGCCTGGTGCCTGTCCGTGAAATGTTGGGCTTCAGAGGTGCGGTCG	946
Oligotype 23	GTGTTGCGTCGTCGCCCAGCCTGGTGCCTGTCCGTGAAATGTTGGGCTTCAGAAGTGCGGTCA	859
Oligotype 24	GTGTTGCGTCGTCGTTCAGCCTGGTGCCTGTCCGTGAAATGTTGGGCTTCAGAAGTGCGGTCG	832
Oligotype 25	GTGTTGCGTCGTCCGCCGTCCCGCGCCCGCCGCGCGAAATGTTAGGCTTCAAAAGTGCGGCCG	609
Oligotype 26	GTGTTGCGTCGTCCGCCGTCCCGCGCCCGCCGCTCGAAATGTTGGGCTTCAAAAGTGCGGCCG	555
Oligotype 27	GTGTTGCGTCGTCCGCTGTTCCGCGCCCGCCGTGCGAAATGTTGGGCTTCAAAAATGTCGCCG	430
Oligotype 28	GTGTTGCGTCGTCCGCCGTCTCGCGCCCGTCGCGTGA-ATGTTAGGCTTCAAGAGTGTGGCCG	410
Oligotype 29	GTGTTGCGTCGCTCGCCGTCCCGCGCCCGCCGCGTGAAATGTTGGGCTTCAAAAGTGCGATCG	370
Oligotype 30	GTGTTGCGTCGTCTGCCGTCCCGCGCCCGCCGCGCGAATTGTTGGGATCCAAGAGTATGGCCG	367
Oligotype 31	GTGTTGCGTCGTCTGCCGTCCCGCGCCCGCCGCGCGAAATATTGGGCTCCAAGAGTATGGCCG	362
Oligotype 32	GTGTTGCGTCGTCCGTCGTCCCGCGCCCGCCGCGCGAAATGTTGGGCTTCAAAAGTGCGGCCG	331
Oligotype 33	GTGTTGCGTCGTCGCCCAGCCTGGTGCCTGTCCGTGAAATGCTGGGCTTCAGAAGTGCGGTCG	325

Oligotype 34	GCGTTGCGTCGTCCGCCGTCCCGCGCCCGCCGCGCGAAATGTTGGGCTTCAAAAATGCGGCCA	320
Oligotype 35	A-GTTGCGTCGTCTCCCGTCCCGCGCCCGCCGCGTGAAATGTTGGGCTTCTAGAGTGTGGCCG	276
Oligotype 36	A-GTTGCGTCGTCTCCCGTCTCGCGCCCGCCGCGCGAAATGTTGGGCTTCTAGAGTGTGGCCG	269
Oligotype 37	GTGTTGCGTCGTCGCCCAGCCTGGTGCCTGTCCGTGAAATGTTGGGCTTCGGAAGTGCGGTCG	265
Oligotype 38	GTGTTGCGTCGTCGCCCAGCCCGGTGCCTGTCCGTGAAATGTTGGGCTTCAGAAGTGCGGTCG	247
Oligotype 39	GTGTCGCGTCGTCGCCCAGCCTGGTGCCTGTCCGTGAAATGTTGGGCTTCAGAAGTGCGGTCG	244
Oligotype 40	GCGTTGCATCGTCCGCCGTCCCGCGCCCGCCGCGCGA-ATGTTGGGCTTCAAAAATGCGGCCG	244
Oligotype 41	GTGTTGCGTCGTCGCCCAGCCTGGCGCCTGTCCGTGAAATGTTGGGCTTCAGAAGTGCGGTCG	235
Oligotype 42	GTGTTGCGTCGTCGCCCAGCCTGGTGCCTGTCCGTGAAATGTCGGGCTTCAGAAGTGCGGTCG	234
Oligotype 43	GTGCTGCGTCGTCGCCCAGCCTGGTGCCTGTCCGTGAAATGTTGGGCTTCAGAAGTGCGGTCG	232
Oligotype 44	GTGTTGCGTCGTCGCCCAGCCTGGTGCCTGTCCGTGAGATGTTGGGCTTCAGAAGTGCGGTCG	229
Oligotype 45	A-GTTGCGTCGTCTCCCGTCCCGCGCCCGCCGCGCGAAATGCTGGGCTTCTAGAGTGTGGCCG	224
Oligotype 46	GTGTTGAGTCGTCCGCTGTTCCACGCCCGCCGTGCGAAATGTTGGGCTTCAATAATGTCGCCG	223
Oligotype 47	GTGTTGCGTCGTCGCCCAGCCTGGTGCCTGTCCGTGAAATGTTGGGCTTCAGAAGCGCGGTCG	204
Oligotype 48	GTGTTGCGTCGTCGCCCAGCCTGGTGCCTGTCCGTGAAATGTTGGGCTGCAGAAGTGCGGTCG	200
Oligotype 49	GTGTTGCGTCGTCGCCCAGCCTGGTGCCTGTCCGTGAAGTGTTGGGCTTCAGAAGTGCGGTCG	182

Table S-8. HLSU unique oligotypes showing the variable positions that were used to define each oligotype (note that this is not the entire sequence) with the number of sequences used to define each oligotype listed in the last column. The unique oligotype sequences are shown with nucleotide variations (relative to Oligotype 1) highlighted in blue to indicate what differences were used to define each oligotype.

HLSU Oligotype	Unique Oligotype	Sequences per oligotype
Oligotype 1	CGCCTCGTTAGCTATGTCCACCATGAAAGCAGGTCCTGCCTAGGTCCGCCTACACCCCTCGCTCCCTTACCGCCGGTGTCCGCGG	28165
Oligotype 2	CGCACCGCCAGTTATGTCCACCATGAAAGCAGGGCCTCTCTAACTCCGGTTAGCCCCGTCAGCGTG--TCGGCCCGCCGCCCTAG	18775
Oligotype 3	CGCATTGCCAGTTATGTCCACCATGAAAGCAGGGCCTCCCTAACTCCGGTTAGCCTTGTCAGCGTG--TCGGCCCGCCGCCCTGG	14468
Oligotype 4	CGCCTCGTTAGCTATGTCCACCATGAAAGCAGGTCCTGCCTAGGTCCGCCTACACCCCTCGCCCCCTTACCGCCGGTGTCCGCGG	7822
Oligotype 5	CGCCTCGTTAGCTATGTCCACCATGAAAGCAGGTCCTGCCTAGGTCCGCCTACACCCCCCGCCCCCTTACCGCCGGTGTCTGCGG	3289

Oligotype 6	CGCCTCGTTAGCTATGTCCACCATGAAAGCAGGTCCTGCCTAGGTTCGCCTACACCCCTCGCCCCCT-ACCGCCAGTGTTCGCGA	3215
Oligotype 7	CGCCTTGTTTGCTATGTCCACCATGAAAGCAGGTCCTGCCTAGGTTCGCCTACACCCCTCGCCCCC--TCCGCCGGTGTCCGCGG	2788
Oligotype 8	CGCCTCGTTAGCTATGTCCACCATGAAAGCAGGTCCTGCCTAGGTTCGCCTACACCCCTGGCCCCCT-ACCGCCAGTGTTCGCGG	2605
Oligotype 9	CGCACCACCAGTTATGTCCACCATGAAAGCAGGGACTCCCTAACTCCGGTCAGCCCCGTCAGCGTG--TTGGCCCGCCGTCCTGG	2545
Oligotype 10	CGCCTCGTTAGCTATGTCCACCATGAAAGCAGGTCCTGCCTAGGTCCGCCTACACTCCCCGCCCCCTTACCGCCGGTGTCTGCGG	2426
Oligotype 11	CGCCTCGTTATCTATGTTCACCATGAAAGCAGGTCCTGCCTAGGTTCGCCTACACCCCTCGCCCCCTTCCCGCCGGTGTCCGCGG	2111
Oligotype 12	CGCCTCGTTAGCTATGTCCACCATGAAAGCAGGTCCTGCCTAGGTTCGCCTACACCCCTCGCCCCCT-ACCGCCGGTGTCCGCGG	2093
Oligotype 13	CGCGTCGTTAGCTATGTCCATCATGAAAGCAGGTCCTGCCTAGGTTCGCCTACACCCCTCGCCCCCTTACCGCCGGTGTCCGCGG	1902
Oligotype 14	CGCATTGCCAGTTATGTCCACCATGAAAGCAGGGCCTCTCTAACTCCGGTTAGCCCCGTCAGCGTG--TCGGCCCGCCGCCCTAG	1800
Oligotype 15	CGCACCGCCAGTTATGTCCACCATGAAAGCAGGGCCTCCCTAACTCCGGTTAGCCTTGTCAGCGTG--TCGGCCCGCCGCCCTGG	1785
Oligotype 16	CGCCTCGTTAGCTATGTCCACCATGAAAGCAGGTCCTGCCTAGGTCCGCCTACACCCCTCGCCCCCTTACCGCCGGCGTCCGCGG	1671
Oligotype 17	CGTCTCGTTAGCTATGTCCACCATGAAAGCAGGTCCTGCCTAGGTCCGCCTACACCCCTCGCTCCCTTACCGCCGGTGTCCGCGG	1271
Oligotype 18	CGCACCGCCAGTTATGTCCACCATGAAAGCAGGGCCTCTCTAACTCCGGTTAGCCCCGTCAGCGTG--TCGGCCCGCCGCCCTGG	1149
Oligotype 19	CGCACCGCCAGTTATGTCCACCATGAAAGCAGGGCCTCTCTAACTCCGGTTGGCCCCGTCAGCGTG--TCGGCCCGCCGCCCTAG	1114
Oligotype 20	CGCATTGCCAGTTATGTCCACCATGAAAGCAGGGCCTCCCTAACTCCGGTTAGCCTTGTCAGCGTG--TCGGCCCGCCGCCCTAG	1051
Oligotype 21	CGCATTGCCAGTTATGTCCACCATGAAAGCAGGGCCTCCCTAACTCCGGTTGGCCTTGTCAGCGTG--TCGGCCCGCCGCCCTGG	967
Oligotype 22	CGCCTCGTTAGCTATGTCCACCATGAAAGCAGGTCCTGCCTAGGTCCGCCTACACCCCTCGCTCCCTTACTGCCGGTGTCCGCGG	945
Oligotype 23	CGCCTCGTTAGCTATGTCCACCATGAAAGCGGGTCTTGCGTAGGTTCGCCTACACACCTCGCCCCCTTACCACCGGTGTCCGCGG	943
Oligotype 24	CGCCTCGTTAGCTATGTTCACCATGAAAGCAGGTCCTGCCTAGGTTCGCCTACACCCCTCGCCCCCTTCCCGCCGGTGTCCGCGG	938
Oligotype 25	CGCCTCGTTAACTATGTCCACCATGAAAGCAGGTCCTGCCTAGGTCCGCCTACACCCCTCGCCCCCTTACCGCCGGTATCCGCGG	832
Oligotype 26	CGCCTCGTTAGCTATGTCCACCATGAAAGCAGGTCCTGCCTAGGTCCGCCGACACCCCTCGCTCCCTTACCGCCGGTGTCCGCGG	775
Oligotype 27	CGCGTCGTTAGCTATGTCCATCATGAAAGCAGGTCCTGCCTAGGTTCGCCTACACCCCTCGCCCCCTTACCGTCGGTGTCCGCGG	715
Oligotype 28	CGCCTCGTTAGCTATGTCCACCATGAAAGCAGGTCCTGCCTAGGTCCGCCTACACCTCCCGCCCCCTTACCGCCGGTGTCTGCGG	678
Oligotype 29	CGCGTCGTTAGCTATGTCCACCATGAAAGCAGGTCCTGCCTAGGTTCGCCTACACCCCTCGCCCCCTTACCGCCGGTGTCCGCGG	660
Oligotype 30	CGCCTCGTTAGCTATGTCCACCATGAAAGCAGGTCCTGCCGAGGTCCGCCTACACCCCTCGCTCCCTTACCGCCGGTGTCCGCGG	639
Oligotype 31	CGCCTCGTTAGCTAGGTCTACCATGAAAACACGTCCTGCCTAGGTTCACCTCCACCCCTCGCCCCCTTACCGCCGATGTCCGCGG	623
Oligotype 32	CGCCTCGTTAGCTATGTCCACCATGAAAGCGGGTCTTGCGTAGGTTCGCCTACACCCCTCGCCCCCTTACCACCGGTGTCCGCGG	576
Oligotype 33	CGCACCGCCAGTTATGTCCACCATGAAAGCAGGGCCTCTCTAACTCCGGTTAGCCCCGTCAGTGTG--TCGGCCCGCCGCCCTAG	555
Oligotype 34	CGCCTTGTTAGCTATGTCCACCATGAAAGCAGGTCTTGCCTAGGTTCGCCTACACCCCTCGCCCCC--TCCGCTGGTGTCCGCGG	512

Oligotype 35	CGCACCGCCAGTTATGTCCACCATGAAAGCAGGGCCTCTCTAACTCCGGTTAGCCTTGTCAGCGTG--TCGGCCCGCCGCCCTGG	490
Oligotype 36	CGCCTTGTTTGCTATGTCCACCATGAAGGCAGGTCCTGCCTAGGTTCGCCTACACCCCTCGCCCCC--TCCGCCGGTGTCCGCGG	475
Oligotype 37	CGCCTCGTTAGCTATGTCCACCATGAAAGCAGGTCCGGCCTAGGTCCGCCTACACCCCTCGCTCCCTTACCGCCGGTGTCCGCGG	471
Oligotype 38	CGCACCGCCAGTTATGTCCACCATGAAAGCAGGGCCTCTCTAACTCCGGTGAGCCCCGTCAGCGTG--TCGGCCCGCCGCCCTAG	465
Oligotype 39	CGCCTTGTTTGCTATGTCCACAATGAAAGCAGGTCCTGCCTAGGTTTGCCTACACCCCTCGCCCCC--TCCGCCGGTGTCCGCGG	442
Oligotype 40	CGCATTGCCAGTTATGTCCACCATGAAAGCAGGGCCTCCCTAACTCCGGTTAGCCTTGTCAGTGTG--TCGGCCCGCCGCCCTGG	414
Oligotype 41	CGCATTGCCAGTTATGTCCACCATGAAAGCAGGGCCTCCCTAACTCCGGTTAGCCCCGTCAGCGTG--TCGGCCCGCCGCCCTAG	395
Oligotype 42	CGCACCACCAGTTATGTCCACCATGAAAGCAGGGCCTCTCTAACTCCGGTTAGCCCCGTCAGCGTG--TCGGCCCGCCGCCCTAG	372
Oligotype 43	CGCATTGCCAGTTATGTCCACCATGAAAGCAGGGCCTCCCTAACTCCGGTGAGCCTTGTCAGCGTG--TCGGCCCGCCGCCCTGG	366
Oligotype 44	CGCCTCGTTAGCTATGTCCACCATGAAAGCAGGTCCTGCCTAGGTCCGTCTACACCCCTCGCCCCCTTACCGCCGGCGTCCGCGG	356
Oligotype 45	CGCCTCGTTAGCTAGGTCTACCATG-AAACAGGTCCTGCCTAGGTTCACCTCCACCCCTCGCCCCCTTACCGCCGATGTCCGCGG	350
Oligotype 46	CGCACCGCCAGTTATGTCCACCATGAAAGCAGGGACTCCCTAACTCCGGTCAGCCCCGTCAGCGTG--TTGGCCCGCCGTCCTGG	336
Oligotype 47	CGCCTCGTTAGCTATGTCCACCATGAAAGCAGGTCCTGCCTAGGTCCGCCTACACCCCTCGCCCCCTTACCGCTGGCGTCCGCGG	335
Oligotype 48	TGCCTCGTTAGCTATGTCCACCATGAAAGCAGGTCCTGCCTAGGTCCGCCTACACCCCCCGCCCCCTTACCGCCGGTGTCTGCGG	298
Oligotype 49	CGCACCACCAGTTATGTCCACCATGAAAGCAGGGCCTCCCTAACTCCGGTTAGCCTTGTCAGCGTG--TCGGCCCGCCGCCCTGG	297
Oligotype 50	CGCCTCGTTAGCTATGTCCACCATGAAAGCAGGTCCTGCCTAGGTCCGCCTACACCCCTCGCTCCCTTACCGCCGGTGTCTGCGG	296
Oligotype 51	CGCCTCGTTAGCTATGTCCACCATGAAAGCAGGTCCTGCCTAGTTCCGCCCACACCCCTCGCCCCCTTACCGCCGGTGTCCGCGG	293
Oligotype 52	CGCCTCGTTAGCTATGTCTACCATGAAAGCAGGTCCTGCCTAGGTCCGCCTACACCCCTCGCTCCCTTACCGCCGGTGTCCGCGG	292
Oligotype 53	CACCTCGTTAGCTATGTCCACCATGAAAGCAGGTCCTGCCTAGGTCCGCCTACACCCCTCGCCCCCTTACCGCCGGCGTCCGCGG	292
Oligotype 54	CGCCTCGTTAGCTATGTCCACCATGAAAGCAGGTCCTACCTAGGTCCGCCTACATCCCTCGCTCCCTTACCGCCGGTGTCCGCGG	290
Oligotype 55	CGCATTGCCAGTTATGTCCACCATGAAAGCAGGGACTCCCTAACTCCGGTCAGCCCCGTCAGCGTG--TTGGCCCGCCGTCCTGG	282
Oligotype 56	CGCCTCGTTAGCTATGTCCACCATGAAAGCAGGTCCTGCCTGGGTCCGCCTACACCCCTCGCTCCC--TCCGCCGGTGTCCGCGG	280
Oligotype 57	CGCCTCGTTAGCTATGTCCACCATGAAAGCAGGTCCTGCCTAGGTCTGCCTACACCCCTCGCCCCCTTACCGCCGGTGTCCGCGG	269
Oligotype 58	CGCCTCGTTAGCTATGTCCACCATGAAAGCAGGTCCTGCCTAGGTCCGCCTACACCCCTCGCCCCCTTACCGCCGGTGTCTGCGG	269
Oligotype 59	CGCCTCGTTAGCTATGTCCACCATAAAAGCAGGTCCTGCCTAGGTCCGCCTACACCTCCCGCCCCCTTACCGCCGGTGTCTGCGG	217
Oligotype 60	CGCACCACCAGTTATGTCCACCATGAAAGCAGGGACTCCCTAACTCCGGTCGGCCCCGTCAGCGTG--TTGGCCCGCCGTCCTGG	214
Oligotype 61	CGCCTCGTTAGCTATGCCCACCATGAAAGCAGGTCCTGCCTAGGTTCGCCTACACCCCTCGCCCCCT-ACCGCCAGTGTTCGCGG	208
Oligotype 62	CGCCTCGTTAGCTATGTCCACCATGAAAGCAGGTCCTGCCTAGGTCCGCCCACACCCCTCGCCCCCTTACCGCCGGTGTCCGCGG	202
Oligotype 63	CGCACCGCCAGTTATGTCCACCATGAAAGCAGGGCCTCGCTAACTCCGGTTAGCCCCGTCAGCGTG--TCGGCCCGCCGCCCTAG	192

Oligotype 64	CGCCTCGTTAGCTATGTCCACCATGAAAGCAGGTCCTGCCTAGGTCCGCCTACACCCCTCGCTCCCTTACCGCCGGTGTTCGCGG	191
Oligotype 65	CGCCTCGTTAGCTATGTCCACCATGAAAGCAGGTCCTGCCTAGGTCCGCCGACACCCCTCGCCCCCTTACCGCCGGTGTCCGCGG	187
Oligotype 66	CGCCTCGTTAGCTATGTCCACCATGAAAGCAGGTCCTGCCTAGGTCCGCCTACACCCCGCGCTCCCTTACCGCCGGTGTCCGCGG	182
Oligotype 67	CGCCTCGTTAGCTATGTTCGCCCCGAAAGCAGGTCCTGCCTAGGTTCGCCTACACCCCTCGCCCCCTTCCCGCCGGTGTCCGCGG	169
Oligotype 68	CGCCTCGTTAGCTATGTCCACCATGAAAGCAGGTCCTGCCGAGGTCCGCCTACACCCCTCGCCCCCTTACCGCCGGTGTCCGCGG	163
Oligotype 69	CGGCTCGTTAGCTATGTCCACCATGAAAGCAGGTCCTGCCTAGGTCCGCCTACATCCCTCGCTCCCTTACCGCCGGTGTCCGCGG	162
Oligotype 70	CGCCTCGTTAGCTATGTCCACCATGAAAGCAGGTCCTGCCTAGGTTCGCCGACACCCCTCGCCCCCT-ACCGCCAGTGTTCGCGA	153
Oligotype 71	CGCCTCGTTAGCTATGTCCACCATGAAAGCAGGTCCTGCCTAGGTCCGCCTACACCCCTCGCCCCCTTACCGCTGGTGTCCGCGG	152
Oligotype 72	CGCACCACCAGTTATGTCCACCATGAAAGCAGGGACTCCCTAACTCCGGTCAGCCCCGTCAGCGTG--TCGGCCCGCCGCCCTAG	148

REFERENCES

Institute of Arctic Biology. (2012). Toolik Field Station: GIS & Remote Sensing. Retrieved from https://toolik.alaska.edu/gis/maps/maps.php?category=general

Longo, W.M., Theroux, S., Giblin, A.E., Zheng, Y., Dillon, J.T., & Huang, Y. (2016). Temperature calibration and phylogenetically distinct distributions for freshwater alkenones: Evidence from northern Alaskan lakes. *Geochimica et Cosmochimica Acta*, 180, 177–196. https://doi.org/10.1016/j.gca.2016.02.019

Longo, W.M., Huang, Y., Yao, Y., Zhao, J., Giblin, A.E., Wang, X., … Shinozuka, Y. (2018). Widespread occurrence of distinct alkenones from Group I haptophytes in freshwater lakes: Implications for paleotemperature and paleoenvironmental reconstructions. *Earth and Planetary Science Letters*, 492, 239–250. https://doi.org/10.1016/j.epsl.2018.04.002

Wharton Jr, R. A., Parker, B. C., Simmons Jr, G. M., Seaburg, K. G., & Love, F. G. (1982). Biogenic calcite structures forming in Lake Fryxell, Antarctica. *Nature*, *295*, 403.

Yang, N., Welch, K. A., Mohajerin, T. J., Telfeyan, K., Chevis, D. A., Grimm, D. A., ... & Johannesson, K. H. (2015). Comparison of arsenic and molybdenum geochemistry in meromictic lakes of the McMurdo Dry Valleys, Antarctica: Implications for oxyanion-forming trace element behavior in permanently stratified lakes. *Chemical Geology*, **404**, 110-125. https://doi.org/10.1016/j.chemgeo.2015.03.029
